# Coumarin Sulfonamides and Amides Derivatives: Design, Synthesis, and Antitumor Activity In Vitro

**DOI:** 10.3390/molecules26040786

**Published:** 2021-02-03

**Authors:** Jing Zhang, Yaling Tan, Guorong Li, Lexian Chen, Minyi Nie, Zhaohua Wang, Hong Ji

**Affiliations:** 1 Key Laboratory of Molecular Target & Clinical Pharmacology and the State Key Laboratory of Respiratory Disease, School of Pharmaceutical Sciences & the Fifth Affiliated Hospital, Guangzhou Medical University, Guangzhou 511436, China; ZhangJingde163yx@163.com (J.Z.); tanyalinglove@163.com (Y.T.); 13423686258@163.com (G.L.); chenlexian666@163.com (L.C.); nieminyi6@163.com (M.N.); 2 School of Basic Medical Sciences, Guangzhou Medical University, Guangzhou 511436, China; gy_wzhh@163.com

**Keywords:** coumarin sulfonamides, coumarin amides, MDA-MB-231, antitumor

## Abstract

Coumarins possesses immeasurable antitumor potential with minimum side effects depending on the substitutions on the basic nucleus, which exhibits great prospects for antitumor drug development. In an attempt to develop novel antitumor candidates, a series of coumarin sulfonamides and amides derivatives were designed and synthetized. The majority of these derivatives showed good cytotoxic activity against MDA-MB-231 and KB cell lines, among which compound **9c** was the most potent against MDA-MB-231 cells, with IC_50_ value of 9.33 μM, comparable to 5-fluorouracil. Further investigation revealed that compound **9c** had versatile properties against tumors, including inhibition of cell migration and invasion as well as inducing apoptosis. Reactive oxygen species (ROS) assay and western blotting analysis suggested that compound **9c** promoted cancer cell apoptosis by increasing ROS levels and upregulating the expression of caspase-3 in MDA-MB-231 cells. These results indicated that compound **9c** could be promising lead compound for further antitumor drug research.

## 1. Introduction

Cancer is a widespread and lethal disease characterized by uncontrolled growth of abnormal cells. It has been expected to be the leading cause of death in the future [1,2]. Although cancer research resulted in a range of innovative and promising therapeutic approaches, the existing drugs for cancer treatment are frequently associated with several drawbacks such as severe side effects, multidrug resistance, poor bioavailability. Therefore, there is an urgent need for the development of more effective and safer anticancer agents.

Coumarins are a wide family of secondary metabolites found in various species of plants but also fungi and microorganisms [3,4], which exhibit diverse pharmacological effects, especially anticancer activity [5,6,7]. Many coumarins and their derivatives have attracted considerable attention as cancer chemopreventive agents and also as cancer therapeutics [8,9,10,11,12,13,14]. Additionally, they have several attractive features, such as low molecular weight, simple structure, high bioavailability, high solubility in most of the organic solvents, and low toxicity, which ensure them a prominent role as lead compounds in drug research and development [15].

**Figure 1:**
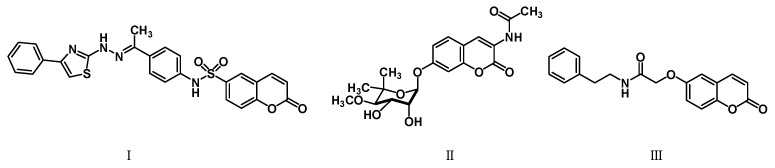
Chemical structures of compounds **I**–**III**.

The promising biological profile of coumarins as antitumor agents and their easy synthetic modifications paved the way for design and synthesis of various coumarin derivatives [16,17]. In the last decade, coumarin derivatives have been increasingly reported to possess antitumor activity through different pharmacological mechanisms, among which coumarin sulfonamides and amides derivatives have attracted great interest because of their potency. Sabt et al. reported a series of novel coumarin-6-sulfonamides as antiproliferative agents [18] and found that compound **I** (Figure 1) was able to induce apoptosis in HepG2 cells by the upregulation of Bax and downregulation of Bcl-2, besides boosting caspase-3 levels. Yu et al. identified compound **II** as a potent inhibitor of the Hsp90 protein-folding machinery [19]. Compound **III** was founded to be a promising LOX and hCA IX inhibitor [20].

In order to discover more potent and selective antitumor agents for further development and as a continuation of our previous studies [21], we designed and synthesized coumarin derivatives bearing sulfonamide and amide moieties. Their cytotoxic activity was screened, and the antitumor effects of the most potent compound on MDA-MB-231 cell line were further investigated.

## 2. Results

### 2.1. Chemical Synthesis

**Scheme 1:**
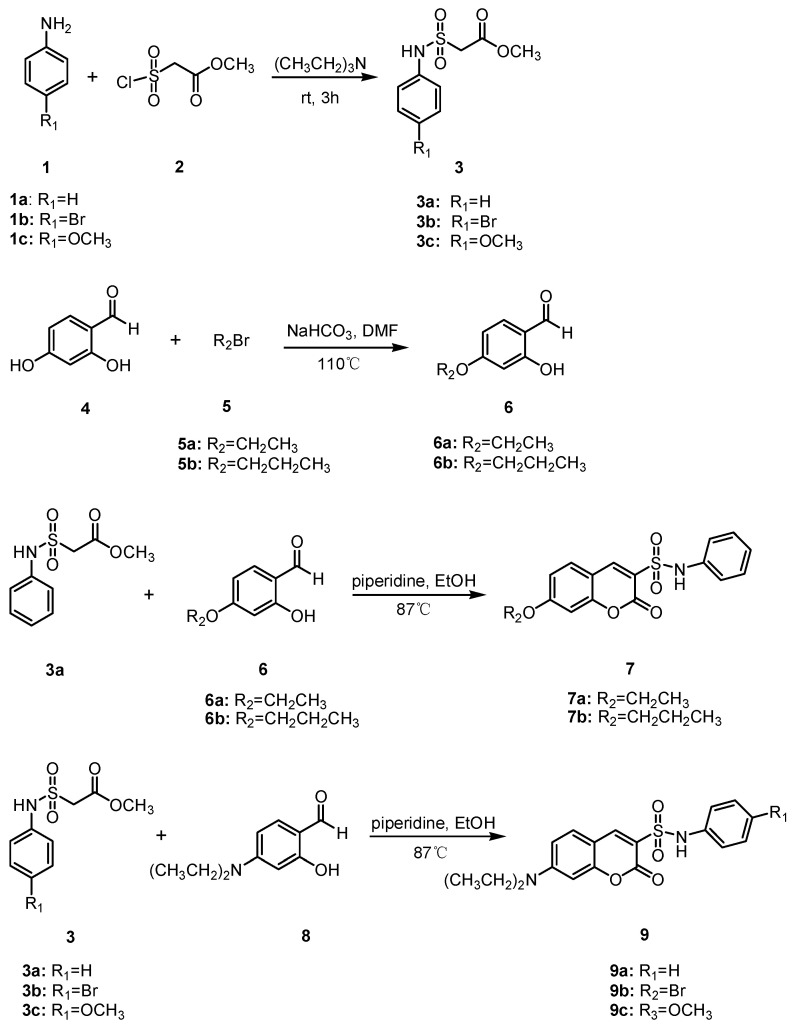
Synthesis of compounds **7a**–**7b** and **9a**–**9c**.

**Scheme 2:**
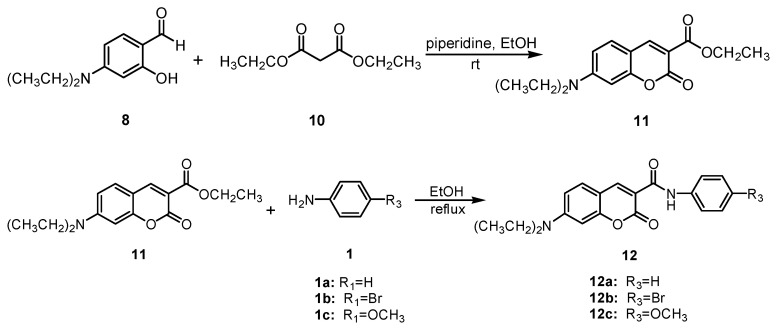
Synthesis of compounds **12a**–**12c**.

The synthesis of coumarin sulfonamides derivatives **7a**–**7b** and **9a**–**9c** were outlined in Scheme 1. Compound **3** were obtained by the sulfonylation of the commercially available aromatic amine **1** with methyl 2-chlorosulfonylacetate **2** in triethylamine at room temperature. The alkylation of 2,4-dihydroxybenzaldehyde **4** with bromide **5** in anhydrous DMF at 110 °C provided compound **6**. The condensation of compound **3a** with substituted salicylaldehyde **6** in anhydrous ethanol in the presence of piperidine gave coumarin sulfonamides **7a**–**7b**. The reaction of compound **3** and 4-(diethylamino) salicylaldehyde **8** in the same condition afforded the desired compound **9**. The coumarin amides derivatives **12a**–**12c** were synthesized as described in Scheme 2. The ethyl coumarin-3-carboxylate **11** was prepared by the condensation of 4-(diethylamino) salicylaldehyde **8** with ethyl malonate **10** in anhydrous ethanol in the presence of piperidine. The ammonolysis of compound **11** with aromatic amines **1** in anhydrous ethanol under reflux led to desired compound **12**.

### 2.2. Cytotoxicity

The in vitro cytotoxicity of the synthesized compounds (Table 1) was evaluated using MTT assay against three human cancer cell lines MDA-MB-231 (human breast cancer cell line), KB (human oral epidermoid carcinoma cell line), and HCT-116 (human colon cancer cell line). 5-Fluorouracil was included as positive control. As shown in Table 2, the majority of the compounds exhibited good inhibitory activities against MDA-MB-231 and KB cell lines, while weak cytotoxicity was observed against HCT-116 cell line except for compounds **9a** and **9b**. Almost all the compounds were more active than 5-fluorouracil against KB cells. In particular, compounds **9b**, **9c** and **12b** showed stronger inhibitory effects than the rest of the compounds. The most potent compound **9c** with the IC_50_ values of 9.33 and 13.66 μM against MDA-MB-231 and KB cells, respectively, was chosen for further investigation.

In addition, it was observed that among coumarin sulfonamides **7a**–**7b** and **9a**–**9c**, 7-diethylaminocoumarin sulfonamides **9a**–**9c** exhibited higher activity than 7-alkoxycoumarin sulfonamides **7a**–**7b**, which implied the diethylamino group at C-7 position improved potency. Coumarin sulfonamides **9a**–**9c** had better cytotoxicity compared to amides derivatives **12a**–**12c**, suggesting the sulfonamide moiety at C-3 position of the coumarin core is favored to the activity. Furthermore, the IC_50_ values of compounds **9a**–**9c** and **12a**–**12c** indicated that substituents on the benzene ring in the sulfonamides or amides had little impact on the potency.

### 2.3. Inhibition of Wound Healing in MDA-MB-231 Cells by Compound ***9c***

**Figure 2:**
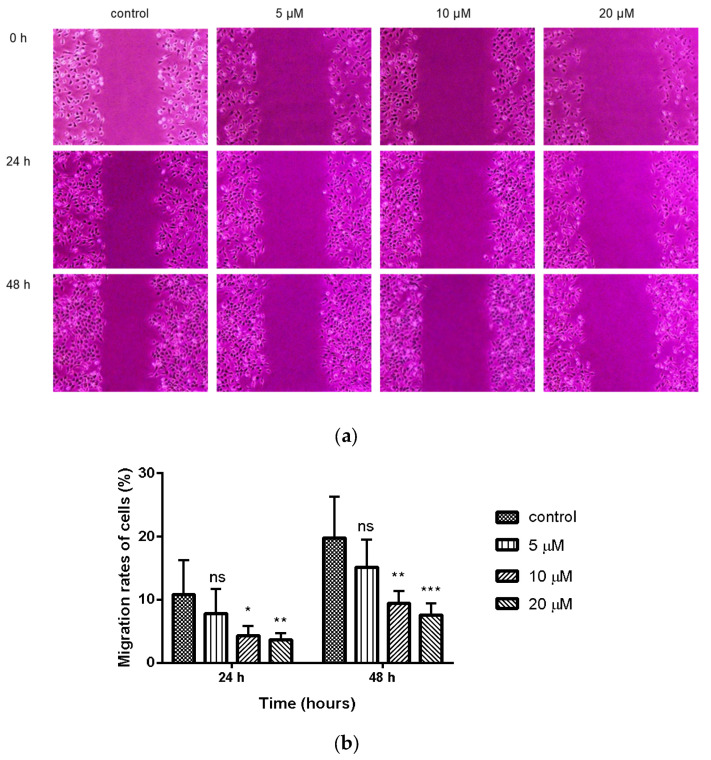
Compound **9c** inhibited wound healing of MDA-MB-231 cells. (**a**) The wound was captured at different time points (0, 24, and 48 h). (**b**) Quantification of the cell scrape results (*n* = 6) by using One-Way ANOVA followed by post-hoc Tukey’s test. *** (*p* < 0.001), ** (*p* < 0.01), * (*p* < 0.05), ns (not significant).

The wound healing assay is a classic and common method used to investigate the effect of antitumor drugs on tumor migration. We next assessed the effect of compound **9c** on the migration of MDA-MB-231 cells with a wound-healing assay. The results showed that the scratch gaps in compound **9c** (10 and 20 μM) treated MDA-MB-231 cells were wider than the control with increased treating time (Figure 2). The cell migration was inhibited, and the wound area was increased compared with the control in a concentration-dependent manner.

### 2.4. Inhibition of Compond ***9c*** on the Invasion of MDA-MB-231 Cells

**Figure 3:**
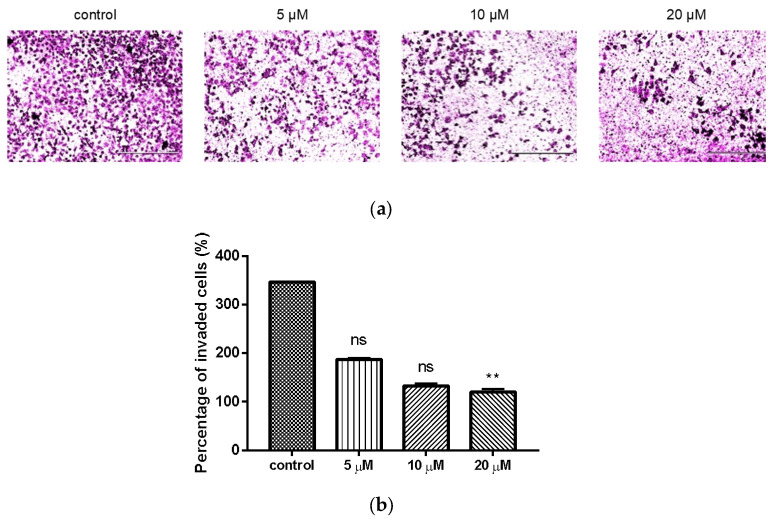
Compound **9c** inhibited invasion of MDA-MB-231 cells. (**a**) Microscope images of the MDA-MB-231 cells that invaded through the matrigel after exposure to different concentrations of compound **9c** for 48 h. Scale bar: 400 μm. (**b**) Quantification of the cell invasion results (*n* = 3) by using Kruskal–Wallis test followed by post-hoc Dunn’s test. ** (*p* < 0.01), ns (not significant).

Transwell invasion assay was used to investigate the effect of compound **9c** on the invasion of MDA-MB-231 cells. Treatment of compound **9c** significantly reduced (*p* < 0.01) the number of invaded cells at the concentration of 20 μM (Figure 3).

### 2.5. Compound ***9c*** Induces Apoptosis in MDA-MB-231 Cells

**Figure 4:**
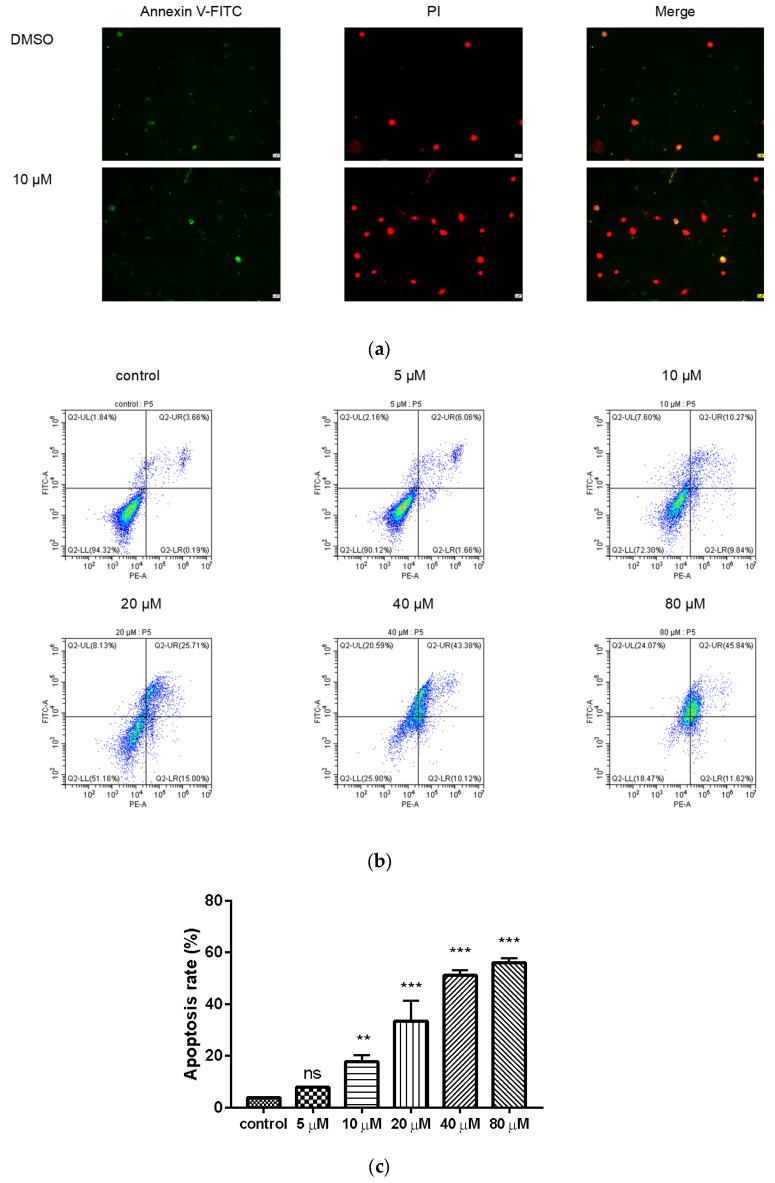
Compound **9c** induced apoptosis of MDA-MB-231 cells. (**a**) Effect of compound **9c** on MDA-MB-231 cells apoptosis morphology. Scale bar: 2 μm. (**b**) Dot plot depicting MDA-MB-231 cells treated with different concentrations of compound **9c** for 48 h. (**c**) Quantification of the cell apoptosis results (*n* = 3) by using One-Way ANOVA followed by post-hoc Tukey’s test. *** (*p* < 0.001), ** (*p* < 0.01), ns (not significant).

To further evaluate whether compound **9c** can induce apoptosis, the Annexin V-FITC/PI double-staining assay was applied with flow cytometer. After treatment with compound **9c** for 48 h, MDA-MB-231 cells were dyed by Annexin V-FITC and showed green fluorescence, which indicates the early phase of apoptosis. The cells dyed by Annexin V-FITC and PI showed red fluorescence inside with green fluorescence outside, which corresponded to the late phase of apoptosis (Figure 4a). The cells treated with compound **9c** (10 μM) had stronger fluorescence intensity compared with the control group, which indicated that compound **9c** promoted apoptosis of MDA-MB-231 cells. Meanwhile, as shown in Figure 4b,c, when treated with different concentrations of compound **9c**, the percentages of apoptotic MDA-MB-231 cells increased from 3.85% in control cells to 7.72%, 20.11%, 40.71%, 53.50%, and 57.46%, respectively. The results implied that compound **9c** could induce the apoptosis of MDA-MB-231 cells in a concentration-dependent manner.

### 2.6. Compound ***9c*** Increases ROS Production in MDA-MB-231 Cells

**Figure 5:**
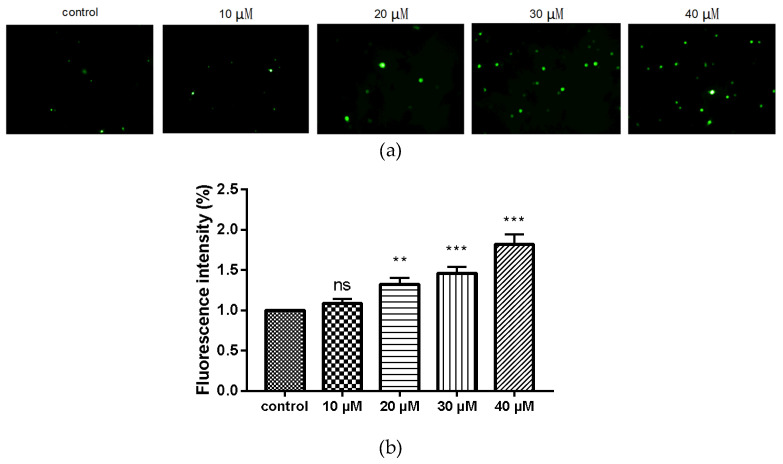
Compound **9c** increased ROS production of MDA-MB-231 cells. (**a**) MDA-MB-231 cells were treated with different concentrations of compound **9c** for 48 h. (**b**) Quantification of the ROS production (*n*= 3) by using One-Way ANOVA followed by post-hoc Tukey’s test. *** (*p* < 0.001), ** (*p* < 0.01), ns (not significant).

We utilized the 2′,7′-dichlorofluorescin diacetate (DCFH-DA) fluorescence probe to further evaluate the changes in intracellular ROS level after treating MDA-MB-231 cells with compound **9c**. Intracellular ROS can oxidize 2′,7′-dichlorofluorescin (DCFH) into the fluorescent 2′,7′-dichlorofluorescein (DCF) whose intensity is proportional to the amount of intracellular ROS. After these cells were subjected to various doses of compound **9c** (10, 20, 30, and 40 μM), the fluorescence intensity increased obviously (Figure 5), revealing that compound **9c** induced intracellular ROS production in concentration-dependent manner.

### 2.7. Western Blot Analysis

**Figure 6:**
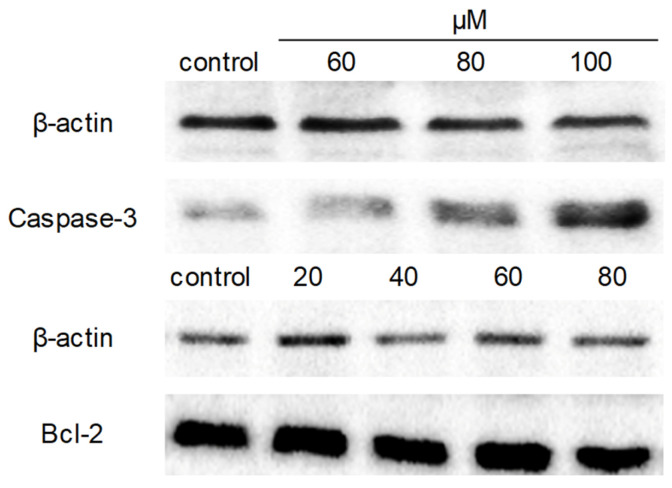
Compound **9c** affected caspase-3 and Bcl-2 expression levels of MDA-MB-231 cells. MDA-MB-231 cells were seeded in 6-well plates and treated with different concentrations of compound **9c** for 48 h. The protein expression levels of caspase-3 and Bcl-2 were evaluated by western blot, β-actin served as a loading control.

Next, Western blot analysis was applied to understand the molecular mechanism of apoptosis induced by compound **9c**. The protein expression levels of caspase-3 and Bcl-2 were detected since caspase-3 played a crucial role in apoptotic pathways by cleaving a variety of key cellular proteins, and Bcl-2 is the well-known anti-apoptotic marker. After exposure of MDA-MB-231 cells to compound **9c**, the expression level of caspase-3 was dramatically increased, while no obvious effect on the expression of Bcl-2 was observed (Figure 6).

## 3. Discussion

Coumarins and their derivatives have been regarded as an important family of anticancer lead compounds, which exhibit diverse effects such as anti-proliferation, anti-invasion, anti-metastasis, anti-angiogenesis and inducing apoptosis. The various mechanisms of their anticancer action have been widely explored [22,23,24,25,26,27,28], including inhibition of carbonic anhydrase, PI3K/AKT/mTOR signaling pathway, microtubule polymerization, monocarboxylate transporters, hypoxia-inducible factor-1, acting on apoptosis proteins, inhibiting tumor multidrug resistance, and regulating ROS.

In this study, coumarin sulfonamide **9c** with the best antiproliferative activity against MDA-MB-231 and KB cell lines were obtained by modification of the basic structure of coumarin. The effects of compound **9c** on the migration and invasion of MDA-MB-231 cells have been further investigated since the ability of cancer cells to migrate and invade into the surrounding tissue is the hallmark of tumor metastasis [29]. Treatment using compound **9c** has shown significant reduction in the percentage of migration and invasion of MDA-MB-231 cells in a concentration-dependent manner.

Apoptosis, an immunogenic programmed cell death, eliminates unhealthy, unwanted cells and maintains surrounding tissue [30]. In cancer, evasion of cell death is one of the major mechanisms for abnormal cells to transform themselves into malignant ones [31]. Thus, drugs that can induce apoptosis, specifically in malignant and metastatic cells, would be the most promising cancer treatment candidates. Annexin V-FITC/PI double-staining assay combined with flow cytometry analysis confirmed that compound **9c** induced apoptosis in MDA-MB-231 cells significantly in a concentration-dependent manner. Most antitumor reagents that can induce apoptosis, including many first-line chemotherapy drugs such as cisplatin, are known to provoke oxidative stress by generating excess ROS, which suggests that abnormal ROS generation is closely associated with apoptosis [32]. Our results of ROS assay showed that ROS levels in MDA-MB-231 cells were significantly increased after treatment with compound **9c**.

The activation of the key apoptotic protein caspase-3 [33] can cleave regulatory proteins essential for cell survival and maintenance as well as poly (ADP-ribose) polymerase (PARP), which is involved in DNA repair and programmed cell death, thereby promoting apoptosis. Bcl-2 as an anti-apoptotic protein has been shown to enhance cell survival by inhibiting apoptosis and enhance the sensitivity of tumor cells to chemotherapy drugs [34,35,36]. Western blotting analysis demonstrated that the expression of caspase-3 was markedly upregulated, whereas little changes in the expression level of Bcl-2 were observed after treating with compound **9c**. These results suggested that compound **9c** induced apoptosis by increasing ROS levels and upregulating caspase-3 expression in MDA-MB-231 cancer cells.

However, the anticancer mechanism of coumarin sulfonamides needs to be further elucidated. Their anticancer potential, security and pharmacokinetic properties in vivo were also left to be investigated. Anyway, this study offers a start point for further elaboration to develop anticancer reagents with coumarin sulfonamide scaffold. More researches which link the anticancer effect, safety, and bioavailability with structure modification are expected to accelerate the development of coumarin derivatives for cancer treatment.

## 4. Materials and Methods

### 4.1. Chemical

#### 4.1.1. Syntheses

All melting points were taken on an X-6 microscope melting point instrument (Bjfuka, Beijing, China) and are uncorrected. Infrared spectra were determined with a TENSORII spectrometer (Bruker, Karlsruhe, Germany). NMR spectra were measured using 300 MHz Bruker AV III spectrometers (Bruker, Karlsruhe, Germany) and 400 MHz JNM-ECZ400S/L1 spectrometers (Varian INOVA, Palo Alto, CA, USA) with tetramethylsilane (TMS) as an internal standard. The following abbreviations were used to designate the multiplicities: s = singlet, d = doublet, t = triplet, q = quartet, m = multiplet, brs = broad singlet. Coupling constant (J) is reported in Hertz (Hz). HR-MS spectra were obtained on Finnigan LCQ and Micromass Auto Spec Ultima-Tof spectrometer (Thermo, Boston, Massachusetts, USA). Analytic thin-layer chromatography (TLC) was performed on precoated silica gel 60 F254 plates (Qingdao Haiyang, Qingdao, Shandong Province, China) using petroleum ether-ethyl acetate as solvent system. All the chemicals were of analytic grade. The detailed preparation procedures of intermediates, byproducts, and target compounds are described below.

#### 4.1.2. General Procedure for the Synthesis of Compounds **3a**–**3c**

Methyl 2-chlorosulfonylacetate **2** (500 mg, 2.90 mmol) was added dropwise to a solution of aromatic amines **1** (4.35 mmol) in Et_3_N (10 mL). The reaction mixture was stirred at room temperature for 3 h. After completion of the reaction, a solution of 10% HCl was added slowly to the mixture to adjust the pH to 7. The mixture was then extracted with DCM (12 mL × 3). The combined organic layer was dried over anhydrous Na_2_SO_4_ and evaporated in a vacuum to give a crude residue which was purified by column chromatography (cyclohexane: ethyl acetate = 15:1–1:1) to yield compounds **3a**–**3c**. The ^1^H-NMR spectra of compounds **3a**–**3c** are in the Supplementary Materials.

*Methyl 2-(N-phenylsulfamoyl)acetate (***3a***)* [37] Yellow solid, yield: 76.3%. ^1^H-NMR (300 MHz, CDCl_3_) *δ* 7.42–7.30 (m, 4H, -C_6_H_5_), 7.29–7.21 (m, 1H, -C_6_H_5_), 7.05 (brs, 1H, -NH-), 3.96 (s, 2H, -CH_2_-COO-), 3.82 (s, 3H, -OCH_3_). HR-MS (ESI): calcd for C_9_H_12_NO_4_S [M + H]^+^ 230.0487, found 230.0481.

*Methyl 2-(N-(4-bromophenyl)sulfamoyl)acetate (***3b***)* [37]. Yellow solid, yield: 86.2%. ^1^H-NMR (300 MHz, CDCl_3_) *δ* 7.52–7.46 (m, 2H, -C_6_H_5_), 7.25–7.18 (m, 2H, -C_6_H_5_), 3.94 (s, 2H, -CH_2_-COO-), 3.82 (s, 3H, -OCH_3_). HR-MS (ESI): calcd for C_9_H_11_BrNO_4_S [M + H]^+^ 307.9592, found 307.9603.

*Methyl 2-(N-(4-methoxyphenyl) sulfamoyl) acetate (***3c****) [37]. Yellow solid powder, yield: 88.2%. ^1^H-NMR (300 MHz, CDCl_3_) *δ* 7.30–7.25 (m, 2H, -C_6_H_5_), 6.91–6.86 (m, 2H, -C_6_H_5_), 6.82 (brs, 1H, -NH-), 3.91 (s, 2H, -CH_2_-COO-), 3.83 (s, 3H, -OCH_3_), 3.80 (s, 3H, -OCH_3_). HR-MS (ESI): calcd for C_10_H_14_NO_5_S [M + H]^+^ 260.0593, found 260.0602.

#### 4.1.3. General Procedure for the Synthesis of Compounds **6a**–**6b**

2,4-dihydroxybenzaldehyde **4** (200 mg, 1.45 mmol) and NaHCO_3_ (240 mg, 2.89 mmol) were dissolved in anhydrous DMF (2 mL), followed by the addition of the bromide **5** (1.45 mmol). The reaction mixture was stirred at 110 °C. After completion of the reaction, the reaction mixture was diluted with DCM (20 mL) and washed with water (10 mL) and saturated brine (10 mL). The organic layer was dried over anhydrous Na_2_SO_4_ and evaporated in a vacuum to give a crude residue which was purified by column chromatography (cyclohexane: ethyl acetate = 200:1) to yield compounds **6a**–**6b**. The ^1^H-NMR spectra of compounds **6a**–**6b** are in the Supplementary Materials.

*4-Ethoxy-2-hydroxybenzaldehyde (***6a***)* [38]. Light yellow liquid, yield: 51.2%. ^1^H-NMR (300 MHz, CDCl_3_) *δ* 11.48 (s, 1H, -CHO), 9.70 (s, 1H, -OH), 7.41 (d, *J* = 8.7 Hz, 1H, -C_6_H_5_), 6.52 (dd, *J* = 8.7, 1.8 Hz, 1H, -C_6_H_5_), 6.40 (d, *J* = 2.4 Hz, 1H, -C_6_H_5_), 4.08 (q, *J* = 7.0 Hz, 2H, -O*CH_2_*CH_3_), 1.43 (d, *J* = 7.2 Hz, 3H, -OCH_2_*CH_3_*). HR-MS (ESI): calcd for C_9_H_11_O_3_ [M + H]^+^ 167.0708, found 167.0704.

*2-hydroxy-4-propoxybenzaldehyde (***6b***)* [38] Light yellow liquid, yield: 57%. ^1^H-NMR (300 MHz, CDCl_3_) *δ* 11.48 (s, 1H, -CHO), 9.70 (d, *J* = 0.4 Hz, 1H, -OH), 7.41 (d, *J* = 8.7 Hz, 1H, -C_6_H_5_), 6.55–6.51 (m, 1H, -C_6_H_5_), 6.41 (d, *J* = 2.2 Hz, 1H, -C_6_H_5_), 3.97 (t, *J* = 7.2 Hz, 2H, -O*CH_2_*CH_2_CH_3_), 1.84 (m, 2H, -OCH_2_*CH_2_*CH_3_), 1.04 (t, *J* = 7.0 Hz, 3H, -OCH_2_CH_2_*CH_3_*). HR-MS (ESI): calcd for C_10_H_13_O_3_ [M + H]^+^ 181.0865, found 181.0862.

#### 4.1.4. General Procedure for the Synthesis of Compounds **7a**–**7b**

Under a N_2_ atmosphere, compound **3a** (0.388 mmol, 89 mg) and compound **6** (0.466 mmol) were dissolved in anhydrous ethanol (2 mL), and piperidine (1.4 mmol, 0.1 mL) was added dropwise. The resulting mixture was stirred at 87 °C. After completion of the reaction, the solvent was removed under reduced pressure and water (10 mL) was added to the residue and then extracted with DCM (10 mL × 3). The combined organic layers were dried over anhydrous Na_2_SO_4_ and evaporated in a vacuum to give a crude residue which was purified by column chromatography (cyclohexane: ethyl acetate = 100:1–5:1) to yield compounds **7a**–**7b**. The NMR spectra of compounds **7a**–**7b** are in the Supplementary Materials.

*7-Ethoxy-2-oxo-N-phenyl-2H-chromene-3-sulfonamide (***7a***)*. Light yellow solid, yield: 50.2%. mp: 204–206°C; IR (KBr) ν_max_ (cm^−1^) 3288, 1722, 1595, 1494; ^1^H-NMR (300 MHz, CDCl_3_) δ 8.35 (s, 1H, -C_6_H_5_), 7.45 (d, *J* = 8.7 Hz, 1H, -C_6_H_5_), 7.30 (d, *J* = 9.2, 7.2 Hz, 4H, -C_6_H_5_), 7.13 (d, *J* = 6.6 Hz, 1H, -C_6_H_5_), 6.88 (d, *J* = 7.7 Hz, 1H, -C_6_H_5_), 6.80 (s, 1H, -C_6_H_5_), 4.27 (q, *J* = 7.2 Hz, 2H, -O*CH_2_*CH_3_), 1.46 (t, *J* = 7.2 Hz, 3H, -OCH_2_*CH_3_*). ^13^C-NMR (75 MHz, DMSO-*d_6_*) δ 164.50 (-COO-), 156.85 (-CH=C), 155.11 (-CH=C), 149.06 (-CH=C), 137.61 (-CH=C), 132.19 (-CH=C), 129.28 (-CH=C), 124.30 (-CH=C), 120.75 (-CH=C), 120.20 (-CH=C), 114.06 (-CH=C), 110.55 (-CH=C), 100.94 (-CH=C), 64.60 (-O*CH_2_*CH_3_), 14.28 (-OCH_2_*CH_3_*). HR-MS (ESI): calcd for C_17_H_16_NO_5_S [M + H]^+^ 346.0749, found 346.0755.

*7-propoxy-2-oxo-N-phenyl-2H-chromene-3-sulfonamide (***7b***).* Light yellow solid, yield: 66.5%. mp: 200–202°C; IR (KBr) ν_max_ (cm^−1^) 3314, 1717, 1615, 1546; ^1^H-NMR (300 MHz, CDCl_3_) *δ* 8.34 (s, 1H, -C_6_H_5_), 7.45 (d, *J* = 8.8 Hz, 1H, -C_6_H_5_), 7.24 (dd, *J* = 9.4, 7.6 Hz, 4H, -C_6_H_5_), 7.13 (t, *J* = 7.4 Hz, 1H, -C_6_H_5_), 6.90 (d, *J* = 8.7 Hz, 1H, -C_6_H_5_), 6.81 (s, 1H, -C_6_H_5_), 4.00 (t, *J* = 6.5 Hz, 2H, -O*CH_2_*CH_2_CH_3_), 1.85 (dd, *J* = 14.0, 7.0 Hz, 2H, -OCH_2_*CH_2_*CH_3_), 1.05 (t, *J* = 7.4 Hz, 3H, -OCH_2_CH_2_*CH_3_*). ^13^C-NMR (75 MHz, DMSO-*d_6_*) *δ* 164.62 (-COO-), 156.81 (-CH=C), 155.09 (-CH=C), 149.03 (-CH=C), 137.17 (-CH=C), 132.14 (-CH=C), 129.13 (-CH=C), 124.05 (-CH=C), 120.75 (-CH=C), 119.92 (-CH=C), 113.98 (-CH=C), 110.52 (-CH=C), 100.91 (-CH=C), 70.19 (-O*CH_2_*CH_2_CH_3_), 21.70 (-OCH_2_*CH_2_*CH_3_), 10.21 (-OCH_2_CH_2_*CH_3_*). HR-MS (ESI): calcd for C_18_H_18_NO_5_S [M + H]^+^ 360.0906, found 360.0897.

#### 4.1.5. General Procedure for the Synthesis of Compounds **9a**–**9c**

Under a N_2_ atmosphere, compound **3** (1 mmol) and 4-(diethylamino) salicylaldehyde **8** (1.2 mmol, 232mg) were dissolved in anhydrous ethanol and piperidine (1.4 mmol, 0.1 mL) was then added dropwise. The resulting mixture was heated under reflux with stirring for 30 min. The solvent was removed under reduced pressure and water (10 mL) was added to the residue, and then extracted with DCM (10 mL × 3). The combined organic layers were dried over anhydrous Na_2_SO_4_ and evaporated in a vacuum to give a crude residue which was purified by column chromatography (cyclohexane: ethyl acetate = 10:1) to yield compounds **9a**–**9c**. The NMR spectra of compounds **9a**–**9c** are in the Supplementary Materials.

*7-(Diethylamino)-2-oxo-N-phenyl-2H-chromene-3-sulfonamide (***9a***).* White solid, yield: 61.3%. mp: 211–213 °C. IR (KBr) ν_max_ (cm^−1^) 3273, 2974, 1708, 1614; ^1^H-NMR (300 MHz, DMSO-*d_6_*) *δ* 10.19 (s, 1H, -NH-), 8.55 (s, 1H, -C_6_H_5_), 7.64 (d, *J* = 9.1 Hz, 1H, -C_6_H_5_), 7.29–7.08 (m, 4H, -C_6_H_5_), 6.98 (t, *J* = 7.2 Hz, 1H, -C_6_H_5_), 6.76 (dd, *J* = 9.1, 2.3 Hz, 1H, -C_6_H_5_), 6.51 (d, *J* = 2.1 Hz, 1H, -C_6_H_5_), 3.44 (q, *J* = 6.9 Hz, 4H, -N*CH_2_*CH_3_), 1.09 (t, *J* = 7.0 Hz, 6H, -NCH_2_*CH_3_*). ^13^C-NMR (75 MHz, DMSO-*d_6_*) *δ* 157.73 (-COO-), 155.75 (-CH=C), 152.99 (-CH=C), 148.47 (-CH=C), 137.62 (-CH=C), 132.05 (-CH=C), 129.04 (-CH=C), 123.68 (-CH=C), 119.59 (-CH=C), 114.85 (-CH=C), 110.13 (-CH=C), 105.96 (-CH=C), 96.01 (-CH=C), 44.41 (-N*CH_2_*CH_3_), 12.23 (-NCH_2_*CH_3_*). HR-MS (ESI): calcd for C_19_H_21_N_2_O_4_S [M + H]^+^ 373.1222, found 373.1229.

*N-(4-bromophenyl)-7-(diethylamino)-2-oxo-2H-chromene-3-sulfonamide (***9b***).* White solid, yield: 59.2%. mp: 192–194°C. IR (KBr) ν_max_ (cm^−1^) 3267, 2976, 1711, 1613; ^1^H-NMR (300 MHz, CDCl_3_) *δ* 8.17 (s, 1H, -C_6_H_5_), 7.38–7.33 (m, 2H, -C_6_H_5_), 7.30 (d, *J* = 9.0 Hz, 1H, -C_6_H_5_), 7.14–7.07 (m, 2H, -C_6_H_5_), 6.61 (dd, *J* = 9.0, 2.5 Hz, 1H, -C_6_H_5_), 6.44 (d, *J* = 2.3 Hz, 1H, -C_6_H_5_), 3.44 (q, *J* = 7.1 Hz, 4H, -N*CH_2_*CH_3_), 1.22 (t, *J* = 7.1 Hz, 6H, -NCH_2_*CH_3_*). ^13^C-NMR (75 MHz, DMSO-*d_6_*) *δ* 157.73 (-COO-), 155.75 (-CH=C), 152.99 (-CH=C), 148.47 (-CH=C), 137.62 (-CH=C), 132.05 (-CH=C), 129.04 (-CH=C), 123.68 (-CH=C), 119.59 (-CH=C), 114.85 (-CH=C), 110.13 (-CH=C), 105.96 (-CH=C), 96.01 (-CH=C), 44.41(-N*CH_2_*CH_3_), 12.23 (-NCH_2_*CH_3_*). HR-MS (ESI): calcd for C_19_H_20_BrN_2_O_4_S [M + H]^+^ 451.0327; found: 451.0334.

*7-(Diethylamino)-N-(4-methoxyphenyl)-2-oxo-2H-chromene-3-sulfonamide (***9c***)*. White solid, yield: 42.8%.mp: 198–199°C. IR (KBr) ν_max_ (cm^−1^) 3271, 2928, 1796, 1615; ^1^H-NMR (300 MHz, CDCl_3_) *δ* 8.12 (s, 1H, -C_6_H_5_), 7.27 (d, *J* = 6.6 Hz, 1H, -C_6_H_5_), 7.16 (d, *J* = 8.8 Hz, 2H, -C_6_H_5_), 6.76 (d, *J* = 8.8 Hz, 2H, -C_6_H_5_), 6.60 (dd, *J* = 9.0, 2.0 Hz, 1H, -C_6_H_5_), 6.46 (s, 1H, -C_6_H_5_), 3.72 (s, 3H, -OCH_3_), 3.42 (q, *J* = 7.1 Hz, 4H, -N*CH_2_*CH_3_), 1.22 (d, *J* = 7.1 Hz, 6H, -NCH_2_*CH_3_*).^13^C-NMR (75 MHz, DMSO-*d_6_*) *δ* 157.70 (-COO-), 156.37 (-CH=C), 155.92 (-CH=C), 152.90 (-CH=C), 148.20 (-CH=C), 131.95 (-CH=C), 129.97 (-CH=C), 123.14 (-CH=C), 115.03 (-CH=C), 114.22 (-CH=C), 110.0 (-CH=C)9, 105.99 (-CH=C), 96.03 (-CH=C), 55.09 (-OCH_3_), 44.40 (-N*CH_2_*CH_3_), 12.25 (-NCH_2_*CH_3_*). HR-MS (ESI): calcd for C_20_H_23_N_2_O_5_S [M + H]^+^: 403.1328; found: 403.1333.

#### 4.1.6. Synthesis of Compound **11**

Under a N_2_ atmosphere, 4-(diethylamino) salicylaldehyde **8** (1 mmol, 193 mg) and diethyl malonate **10** (2 mmol, 320 mg) were dissolved in anhydrous ethanol (10 mL) and piperidine (1.4 mmol, 0.1 mL) was added dropwise. The reaction mixture was stirred at room temperature for 3 h. 10% HCl was then added into the reaction mixture to adjust the pH to 8. The mixture was then extracted with ethyl acetate (10 mL × 3). The combined organic layer was dried over anhydrous Na_2_SO_4_ and evaporated in a vacuum to give a crude residue which was purified by column chromatography (petroleum ether: ethyl acetate = 8:1) to yield compound **11**. The NMR spectra of compound **11** are in the Supplementary Materials.

*Ethyl 7-(diethylamino)-2-oxo-2H-chromene-3-carboxylate (***11***)* [39]. Yellow solid; yield 75%. ^1^H-NMR (400 MHz, CDCl_3_) *δ* 8.40 (s, 1H, -C_6_H_5_), 7.33 (d, *J* = 8.9 Hz, 1H, -C_6_H_5_), 6.58 (dd, *J* = 8.9, 2.4 Hz, 1H, -C_6_H_5_), 6.43 (d, *J* = 2.3 Hz, 1H, -C_6_H_5_), 4.34 (q, *J* = 7.1 Hz, 2H, -O*CH_2_*CH_3_), 3.42 (q, *J* = 7.1 Hz, 4H, -N*CH_2_*CH_3_), 1.37 (t, *J* = 7.1 Hz, 3H, -OCH_2_*CH_3_*), 1.21 (t, *J* = 7.1 Hz, 6H, -NCH_2_*CH_3_*). ^13^C-NMR (100 MHz, CDCl_3_) *δ* 164.37 (-COO-), 158.54 (-COO-), 152.96 (-CH=C), 149.33 (-CH=C), 131.14 (-CH=C), 109.62 (-CH=C), 108.97 (-CH=C), 107.76 (-CH=C), 96.78 (-CH=C), 61.25 (-O*CH_2_*CH_3_), 45.19 (-N*CH_2_*CH_3_), 14.49 (-OCH_2_*CH_3_*), 12.52 (-NCH_2_*CH_3_*). HR-MS (ESI): calcd for C_16_H_20_NO_4_ [M + H]^+^ 290.1392, found 290.1386.

#### 4.1.7. General Procedure for the Synthesis of Compounds **12a**–**12c**

Compound **11** (1 mmol, 289 mg) and aromatic amines **1** (1.2 mmol) were dissolved in ethanol (10 mL). The reaction mixture was heated under reflux. After completion of the reaction, the solvent was evaporated in a vacuum followed by the addition of water (10 mL) and 10% HCl to adjust the pH to 8. The mixture was then extracted with ethyl acetate (20 mL × 3). The combined organic layer was dried over anhydrous Na_2_SO_4_ and evaporated in a vacuum to give a crude residue which was purified by column chromatography (petroleum ether: ethyl acetate = 5:1–1:1) to yield compounds **12a**–**12c**. The NMR spectra of compounds **12a**–**12c** are in the Supplementary Materials.

*7-(diethylamino)-2-oxo-N-phenyl-2H-chromene-3-carboxamide (***12a***).* Yellow solid; yield 60%; mp: 156.1–160.0 °C. IR (KBr) ν_max_ (cm^−1^) 3125, 1699, 1605. ^1^H-NMR (400 MHz, CDCl_3_) *δ* 10.87 (s, 1H, -NH-), 8.80 (s, 1H, -C_6_H_5_), 7.74 (dd, *J* = 8.5, 0.9 Hz, 2H, -C_6_H_5_), 7.47 (d, *J* = 9.0 Hz, 1H, -C_6_H_5_), 7.33–7.38 (m, 2H, -C_6_H_5_), 7.12 (t, *J* = 7.4 Hz, 1H, -C_6_H_5_), 6.67 (dd, *J* = 9.0, 2.5 Hz, 1H, -C_6_H_5_), 6.53 (d, *J* = 2.4 Hz, 1H, -C_6_H_5_), 3.47 (q, *J* = 7.2 Hz, 4H, -N*CH_2_*CH_3_), 1.25 (t, *J* = 7.2 Hz, 6H, -NCH_2_*CH_3_*). ^13^C-NMR (100 MHz, CDCl_3_) *δ* 163.20 (-COO-), 161.21 (-CONH-), 157.86 (-CH=C), 152.89 (-CH=C), 148.59 (-CH=C), 138.43 (-CH=C), 131.42 (-CH=C), 129.04 (-CH=C), 124.20 (-CH=C), 120.46 (-CH=C), 110.27 (-CH=C), 108.66 (-CH=C), 96.68 (-CH=C), 45.26 (-N*CH_2_*CH_3_), 12.55 (-NCH_2_*CH_3_*). HR-MS (ESI): calcd for C_20_H_21_N_2_O_3_ [M + H]^+^: 337.1552; found: 337.1561.

*N-(4-bromophenyl)-7-(diethylamino)-2-oxo-2H-chromene-3-carboxamide (***12b***)*. Yellow solid; yield 59%; mp: 231.5–235.6 °C. IR (KBr) ν_max_ (cm^−1^) 3431, 1685, 1617. ^1^H-NMR (400 MHz, CDCl_3_) *δ* 8.38 (s, 1H, -C_6_H_5_), 7.48 (d, *J* = 8.7 Hz, 2H, -C_6_H_5_), 7.15 (d, *J* = 8.8 Hz, 1H, -C_6_H_5_), 7.10 (d, *J* = 8.7 Hz, 2H, -C_6_H_5_), 6.25 (dd, *J* = 8.8, 2.5 Hz, 1H, -C_6_H_5_), 6.18 (d, *J* = 2.5 Hz, 1H, -C_6_H_5_), 3.40 (q, *J* = 7.1 Hz, 4H, -N*CH_2_*CH_3_), 1.21 (t, *J* = 7.1 Hz, 6H, -NCH_2_*CH_3_*). ^13^C-NMR (100 MHz, CDCl_3_) *δ* 162.90 (-COO-), 159.82 (-CONH-), 156.78 (-CH=C), 150.96 (-CH=C), 147.07 (-CH=C), 132.87 (-CH=C), 131.22 (-CH=C), 121.47 (-CH=C), 107.93 (-CH=C), 102.89 (-CH=C), 96.66 (-CH=C), 43.61 (-N*CH_2_*CH_3_), 11.68 (-NCH_2_*CH_3_*). HR-MS (ESI): calcd for C_20_H_20_BrN_2_O_3_ [M + H]^+^: 415.0657; found: 415.0646.

*7-(diethylamino)-N-(4-methoxyphenyl)-2-oxo-2H-chromene-3-carboxamide (***12c***).* Yellow solid; yield 59%; mp: 189.8–194.0 °C. IR (KBr) ν_max_ (cm^−1^) 3260, 1687,1588. ^1^H-NMR (400 MHz, CDCl_3_) *δ* 8.36 (s, 1H, -C_6_H_5_), 7.20 (d, *J* = 8.8 Hz, 2H, -C_6_H_5_), 7.14 (d, *J* = 8.5 Hz, 1H, -C_6_H_5_), 6.92 (d, *J* = 8.8 Hz, 2H, -C_6_H_5_), 6.24 (d, *J* = 7.6 Hz, 2H, -C_6_H_5_), 3.82 (s, 3H, -OCH_3_), 3.40 (q, *J* = 7.1 Hz, 4H, -N*CH_2_*CH_3_), 1.20 (t, *J* = 7.1 Hz, 6H, -NCH_2_*CH_3_*). ^13^C-NMR (100 MHz, CDCl_3_) *δ* 163.14 (-COO-), 157.48 (-CONH-), 156.78 (-CH=C), 150.87 (-CH=C), 132.70 (-CH=C), 123.34 (-CH=C), 122.45 (-CH=C), 120.53 (-CH=C), 113.52 (-CH=C), 107.84 (-CH=C), 102.85 (-CH=C), 96.90 (-CH=C), 54.52 (-OCH_3_), 43.62 (-N*CH_2_*CH_3_), 11.70 (-NCH_2_*CH_3_*). HR-MS (ESI): calcd for C_21_H_23_N_2_O_4_ [M + H]^+^: 367.1658; found: 367.1666.

### 4.2. Biology

#### 4.2.1. Cell Culture

MDA-MB-231 (ATCC^®^ HTB-26™), KB (ATCC^®^ CCL-17™) and HCT-116 (ATCC^®^ CCL-247™) cells were purchased from the American Type Culture Collection (ATCC, Manassas, VA, USA). Cells were cultured in Dulbecco’s Modified Eagle’s Medium (DMEM) media with 10% fetal bovine serum (FBS), 1% penicillin/streptomycin at 37 °C in a 5% CO_2_ incubator.

#### 4.2.2. MTT Assay

4 × 10^3^ cells were seeded in each well of the 96-well plate in triplicates followed by 24 h of incubation. The cells were treated with various concentrations of compounds (0.78, 1.56, 3.12, 6.25, 12.50, 25.00, and 50.00 μM). After a 72-h incubation, 3-(4,5-dimethylthiazol-2-yl)-2,5-diphenyltetrazolium bromide (MTT) were added, and the cells were incubated for another 4 h prior to the addition of DMSO. Absorbance was recorded at a wavelength of 540 nm with a reference length of 655 nm, and the results shown were collected from three independent experiments. The IC_50_ was determined by using GraphPad Prism software version 7.0 and nonlinear regression (curve fit).

#### 4.2.3. Cell Migration Assay

5 × 10^5^ MDA-MB-231 cells per well were cultured in a 6-well plate and allowed to reach 80% confluency for 12 h. The monolayer was then scratched using a 200 µL pipet tip to produce a wound. The culture medium was removed, and each plate was washed with phosphate buffer saline (PBS) and treated with different concentrations of compound **9c** (5, 10, and 20 μM) for 24 and 48 h, respectively. Images were captured at the identified time point (0, 24, and 48 h) and processed with the ImageJ software.

#### 4.2.4. Analysis of Cell Invasion

For the assay, 24-well transwell chambers coated with 45 µL matrigel were used. In brief, 6 × 10^4^ MDA-MB-231 cells per well were seeded in the upper part of the insert filled with 200 µL of DMEM without FBS, and for the lower part, the chamber was filled with 600 µL DMEM containing 10% FBS. The cells were allowed to grow for 48 h followed by treatment with different concentrations of compound **9c** (5, 10, and 20 μM). The invaded cells were fixed with paraformaldehyde for 2 h and stained with crystal violet solution (0.2%) for 1 h, and non-invading cells in the top chambers of the transwell plates were scraped away by cotton swabs. Every insert was imaged at 10× magnification and analyzed using ImageJ software.

#### 4.2.5. Apoptosis Analysis and Morphology Observation

5 × 10^5^ MDA-MB-231 cells per well were seeded in a 6-well plate for 12 h and were treated with different concentrations of compound **9c** (5, 10, 20, 40, and 80 μM) for 48 h. After treatment, the cells were harvested, washed twice with ice-cold PBS and resuspended in 500 µL of 1× Annexin V binding buffer and then mixed with 5 µL of Annexin V-FITC and 5 µL of PI solution at room temperature in the dark for 10 min. Then, one part of the cells was immediately analyzed with a flow cytometer. The other part was washed twice with PBS, and the cells were photo-graphed by fluorescence microscope.

#### 4.2.6. Intracellular ROS Level Detection

In 6-well plates, 2 × 10^5^ MDA-MB-231 cells were plated and treated with different concentrations (0, 10, 20, 30, and 40 μM) of compound **9c** for 48 h. ROS assay kit (Beyotime) was used to measure intracellular ROS level. DCFH-DA diluted with serum-free medium with 1:1000 was added into the cells at 37 °C for 20 min. The cells were collected after washing three times with serum-free medium. Images were acquired using an inverted fluorescence microscope, and the fluorescence intensity was detected at an excitation wavelength of 488 nm and an emission wavelength of 525 nm with microplate reader.

#### 4.2.7. Western Blotting

For Western blotting analyses, the collected cells were rinsed in PBS and lysed in sodium dodecyl sulfate (SDS) lysis buffer. Equal amounts of protein were separated on 10% SDS-polyacrylamide gels, transferred to polyvinylidene fluoride (PVDF) membranes, and blocked with 5% nonfat dry milk in tris-buffered saline and tween 20 (TBST) for 1 h. The membranes were then incubated with primary antibodies (caspase-3, Bcl-2 and β-actin) overnight at 4 °C. After being washed three times with TBST for 10 min, the membranes were incubated with appropriate secondary antibodies in TBST for 1 h. After several washes of TBST, the blots were developed by enhanced chemiluminescence (ECL) solution.

#### 4.2.8. Statistical Analysis

GraphPad Prism software version 7.0 was used to determine the differences between groups. Values with a *p*-value less than 0.05 are considered as significant and statistical significance is represented as **** (*p* < 0.0001), *** (*p* < 0.001), ** (*p* < 0.01), * (*p* < 0.05), ns (not significant).

## 5. Conclusions

A novel series of coumarin sulfonamides and amides derivatives were designed and synthesized. Compound **9c** was found to be the most potent derivative, which displayed favorable antiproliferative activities toward MDA-MB-231 cells and inhibited migration and invasion and induced apoptosis in MDA-MB-231 cells. Furthermore, it was verified that compound **9c** promoted MDA-MB-231 cell apoptosis by increasing intracellular ROS levels and upregulating the expression of caspase-3. This work presents information that is helpful for the design and synthesis of new coumarin derivatives as potential antitumor drug candidates.

## Figures and Tables

**Table 1:** The structures of coumarin sulfonamides and coumarin amides derivatives.

Compd	Structure	Compd	Structure
**7a**	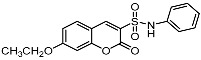	**9c**	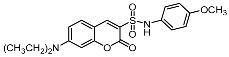
**7b**	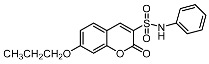	**12a**	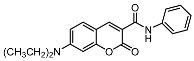
**9a**	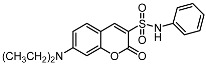	**12b**	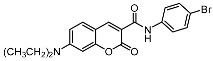
**9b**	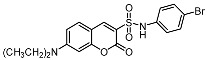	**12c**	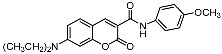

**Table 2:** Inhibition of cancer cells proliferation by compounds **7a**–**7b**, **9a**–**9c** and **12a**–**12c**.

Compd	IC_50_ (μM)		
	MDA-MB-231	KB	HCT-116
**7a**	>100	79.46 ± 1.62	>100
**7b**	>100	20.21 ± 0.42	>100
**9a**	>100	34.94 ± 2.83	15.51 ± 1.59
**9b**	17.13 ± 0.68	10.78 ± 0.13	17.26 ± 2.00
**9c**	9.33 ± 1.81	13.66 ± 1.33	>100
**12a**	13.33 ± 1.26	>100	>100
**12b**	11.63 ± 0.98	14.22 ± 1.64	>100
**12c**	17.01 ± 1.04	97.14 ± 2.43	>100
**5-Fu**	8.59 ± 0.52	94.15 ± 2.76	15.28 ± 0.31

## Data Availability

The data presented in this study are available in Supplementary Materials.

## Supplementary Materials

The following are available online. ^1^H- and ^13^C-NMR spectra of all compounds.

## Sample Availability

Samples of the compounds are available from the authors.
