# Supplementary material for: Coumarin Sulfonamides and Amides Derivatives: Design, Synthesis, and Antitumor Activity In Vitro
Source: Molecules. 2021 Feb 3;26(4):786. doi: 10.3390/molecules26040786 (PMC7913302; doi:10.3390/molecules26040786)
Supplement: Supplementary file 1 [file molecules-26-00786-s001.pdf]

Coumarin Sulfonamides and Amides Derivatives: Design, Synthesis, and Antitumor Activity *in Vitro*

Jing Zhang [1], Yaling Tan [1], Guorong Li [1], Lexian Chen [1], Minyi Nie [1], Zhaohua Wang [2] and Hong Ji [1,*]

[1] Key Laboratory of Molecular Target & Clinical Pharmacology and the State Key Laboratory of Respiratory Disease, School of Pharmaceutical Sciences & the Fifth Affiliated Hospital, Guangzhou Medical University, Guangzhou, 511436, P. R. China; ZhangJingde163yx@163.com (J.Z.); tanyalinglove@163.com (Y.T.); 13423686258@163.com (G.L.); chenlexian666@163.com (L.C.); nieminyi6@163.com

[2] School of Basic Medical Sciences, Guangzhou Medical University, Guangzhou, 511436, P. R. China; gy_wzhh@163.com

* Correspondence: dljih@126.com

Table of Contents

Copies of ^{1}H NMR spectra of compounds **3a-3c**..2

Copies of ^{1}H NMR spectra of compounds **6a-6b** 3

Copies of ^{1}H and ^{13}C NMR spectra of compound **7a-7b**...................................4

Copies of ^{1}H and ^{13}C NMR spectra of compound **9a-9c**...................................6

Copies of ^{1}H and ^{13}C NMR spectra of compound **11**..9

Copies of ^{1}H and ^{13}C NMR spectra of compound **12a-12c**.............................10

^{1}H NMR (300 MHz, $CDCl_3$) of Compound **3a**

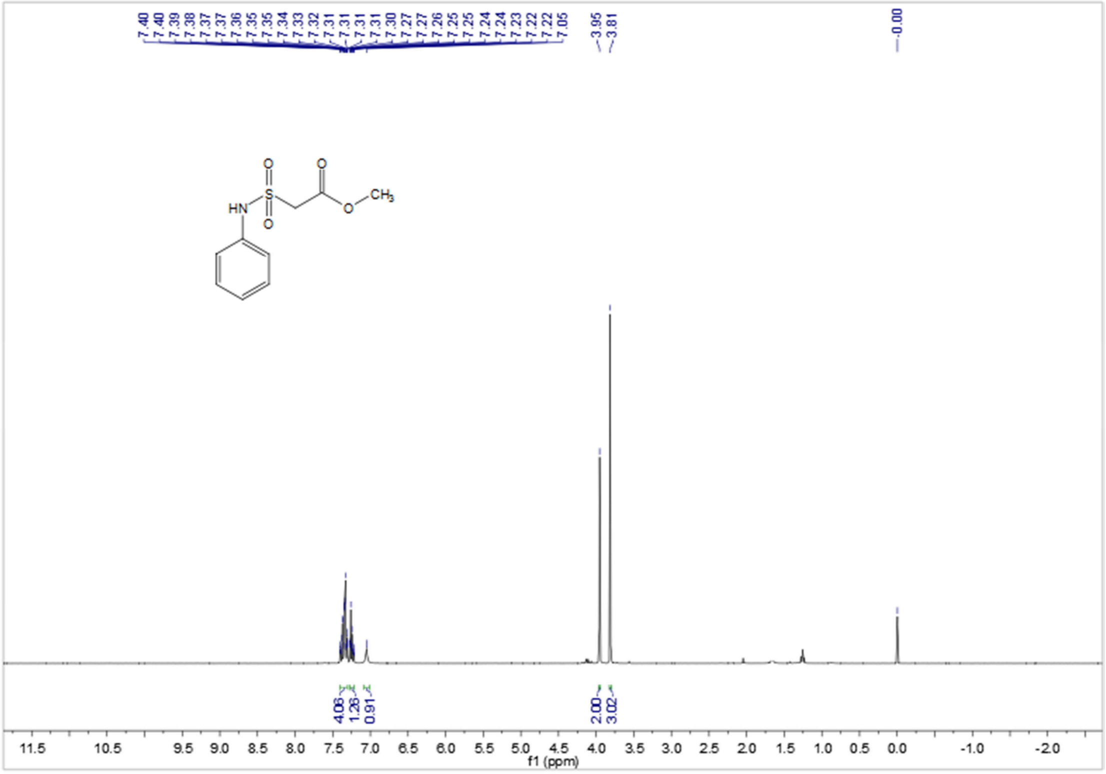

^{1}H NMR (300 MHz, $CDCl_3$) of Compound **3b**

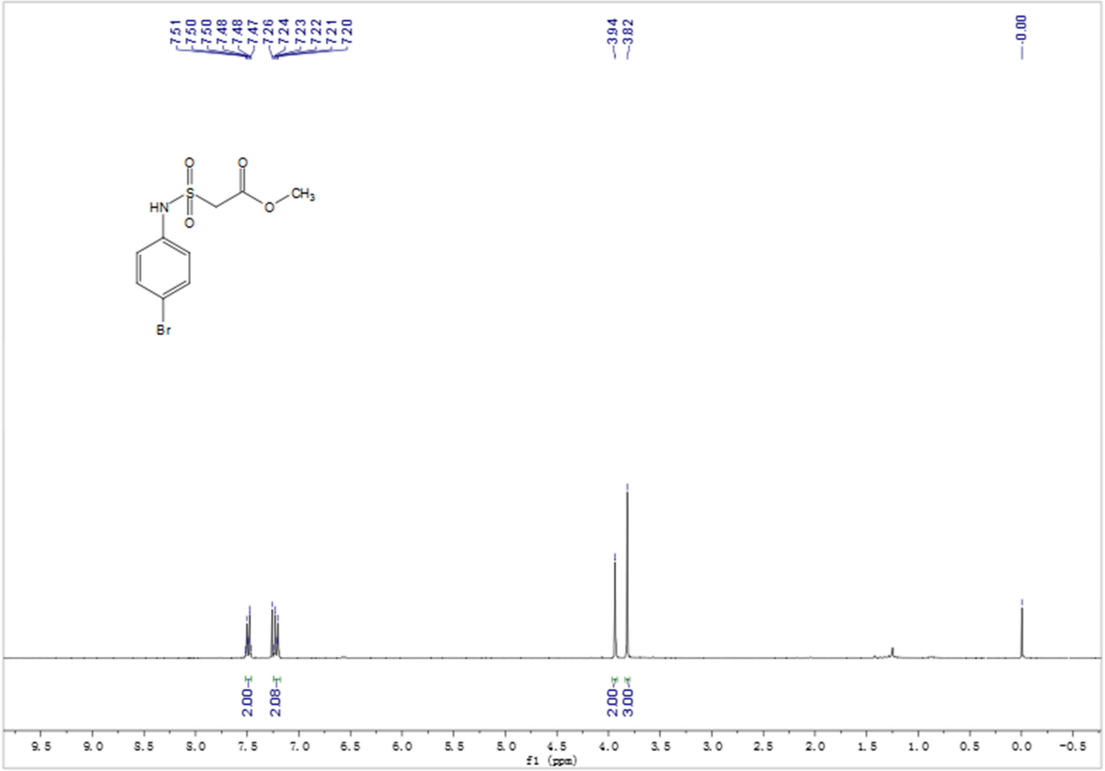

^{1}H NMR (300 MHz, $CDCl_3$) of Compound **3c**

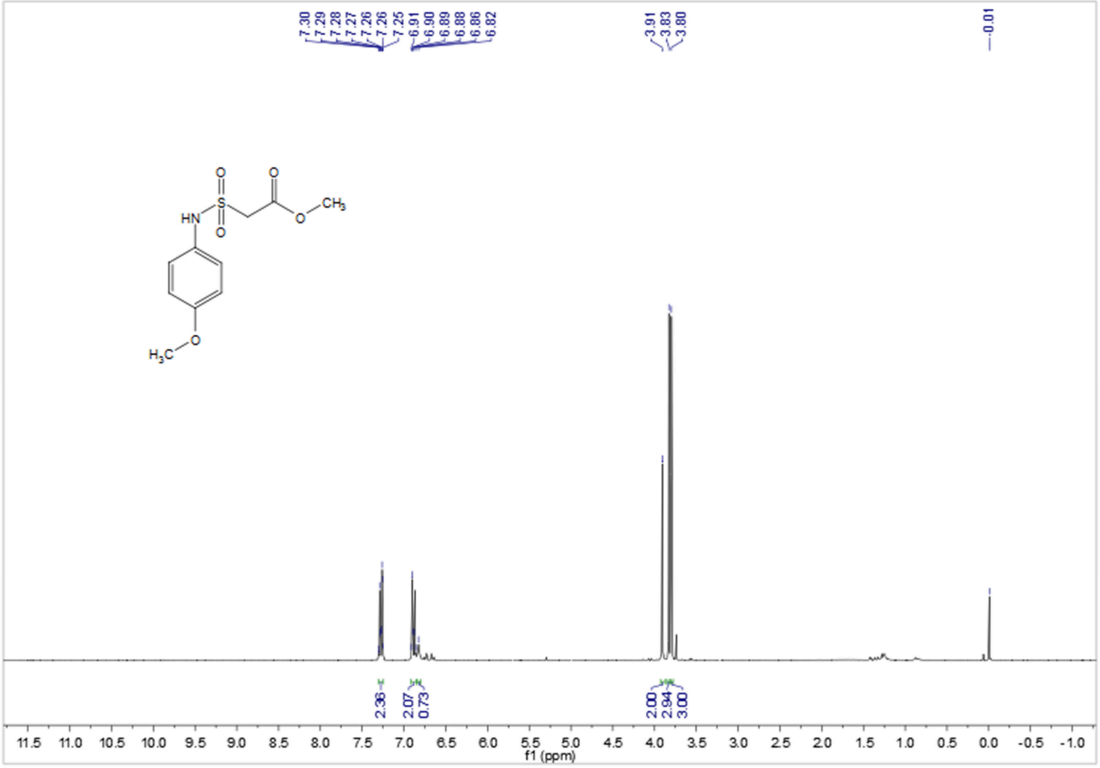

^{1}H NMR (300 MHz, $CDCl_3$) of Compound **6a**

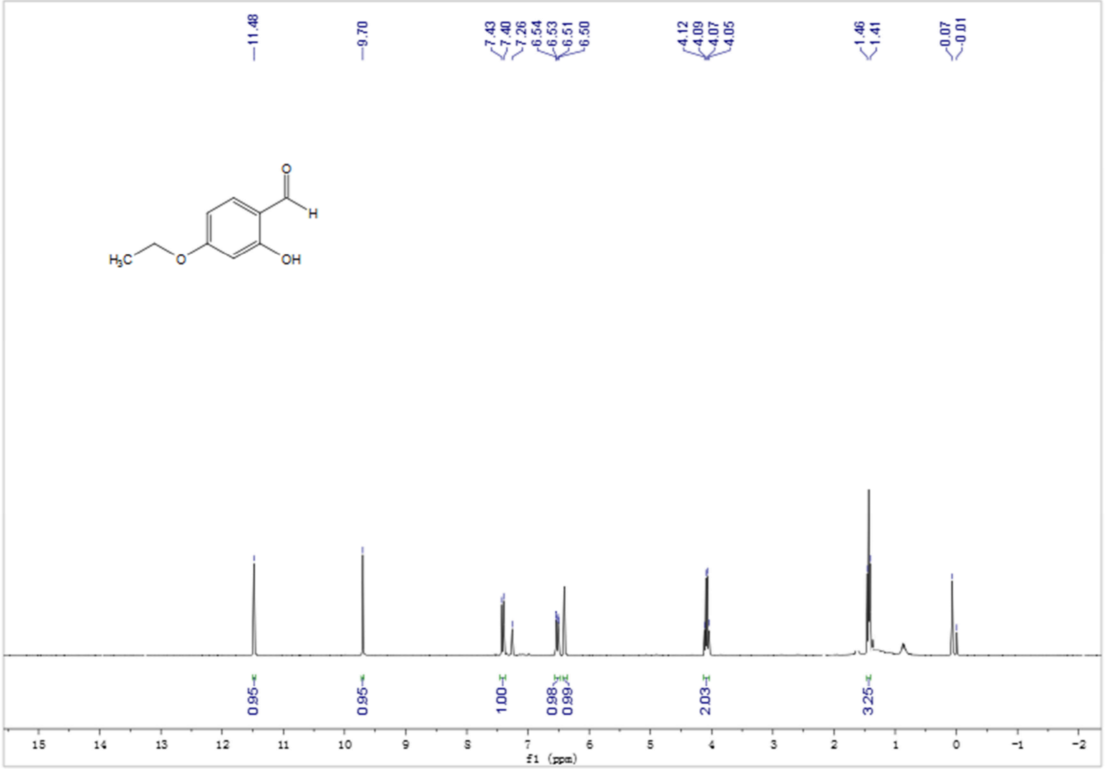

^{1}H NMR (300 MHz, $CDCl_3$) of Compound **6b**

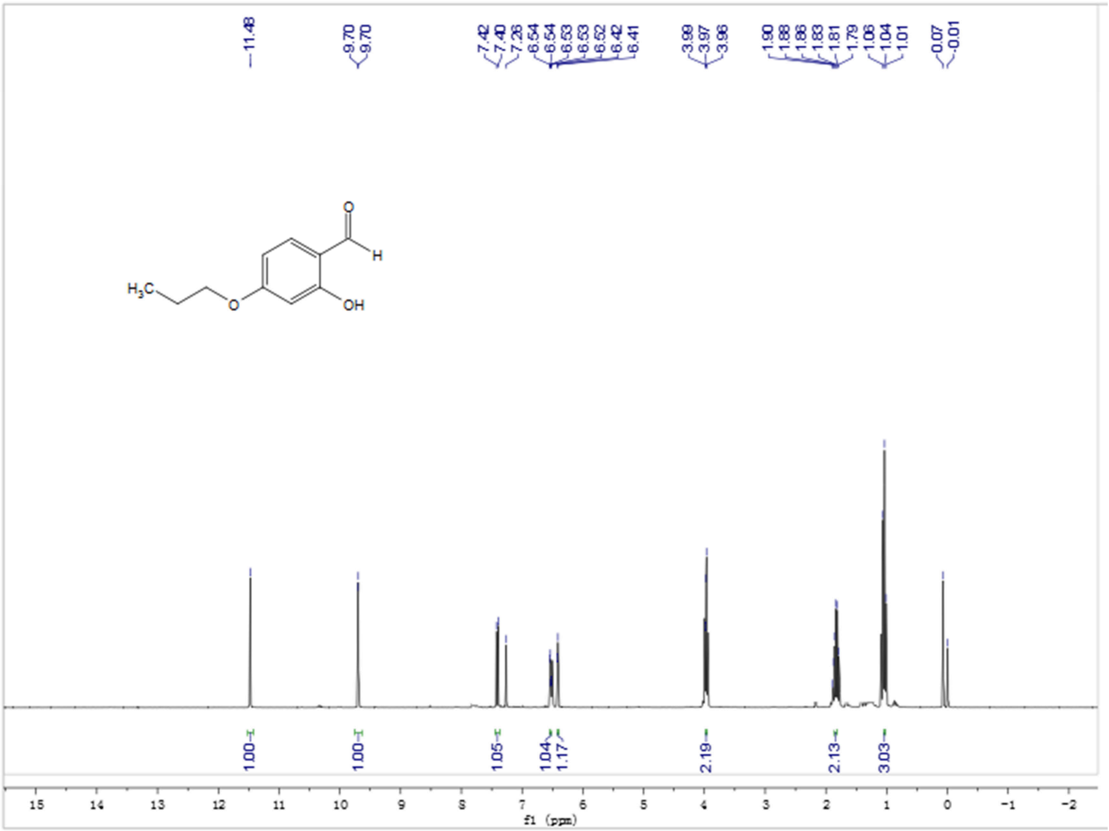

^{1}H NMR (300 MHz, $CDCl_3$) of Compound **7a**

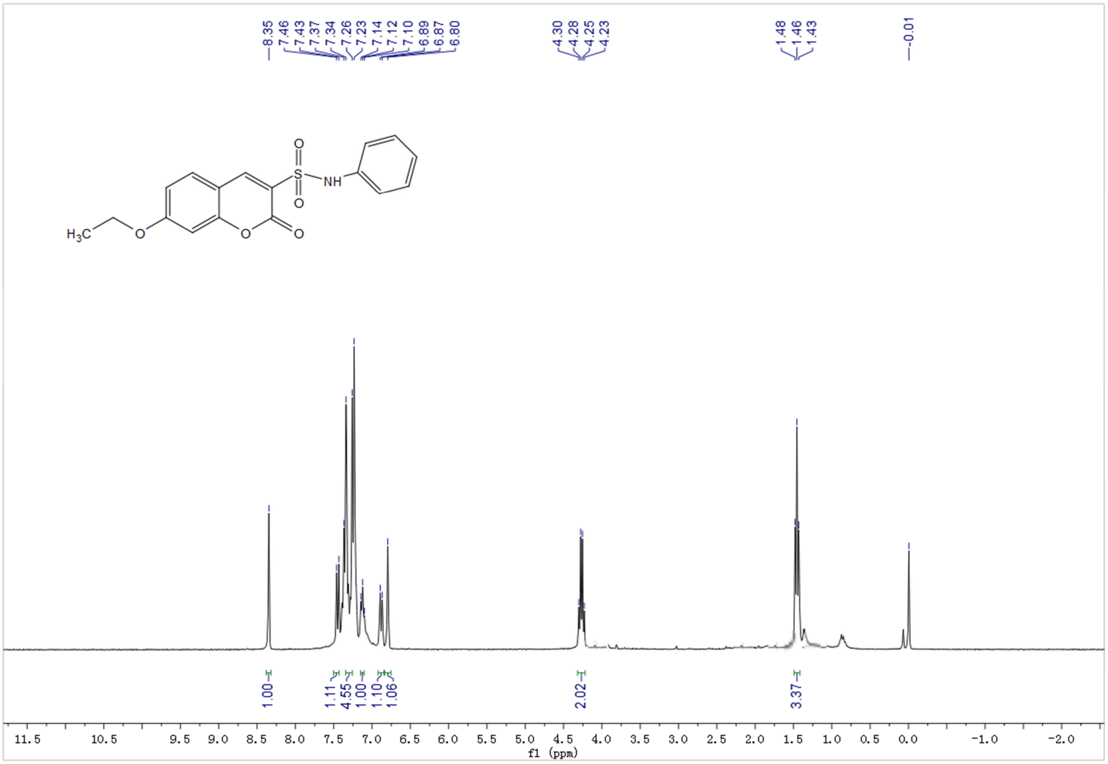

^{13}C NMR (75 MHz, DMSO-d_6) of Compound **7a**

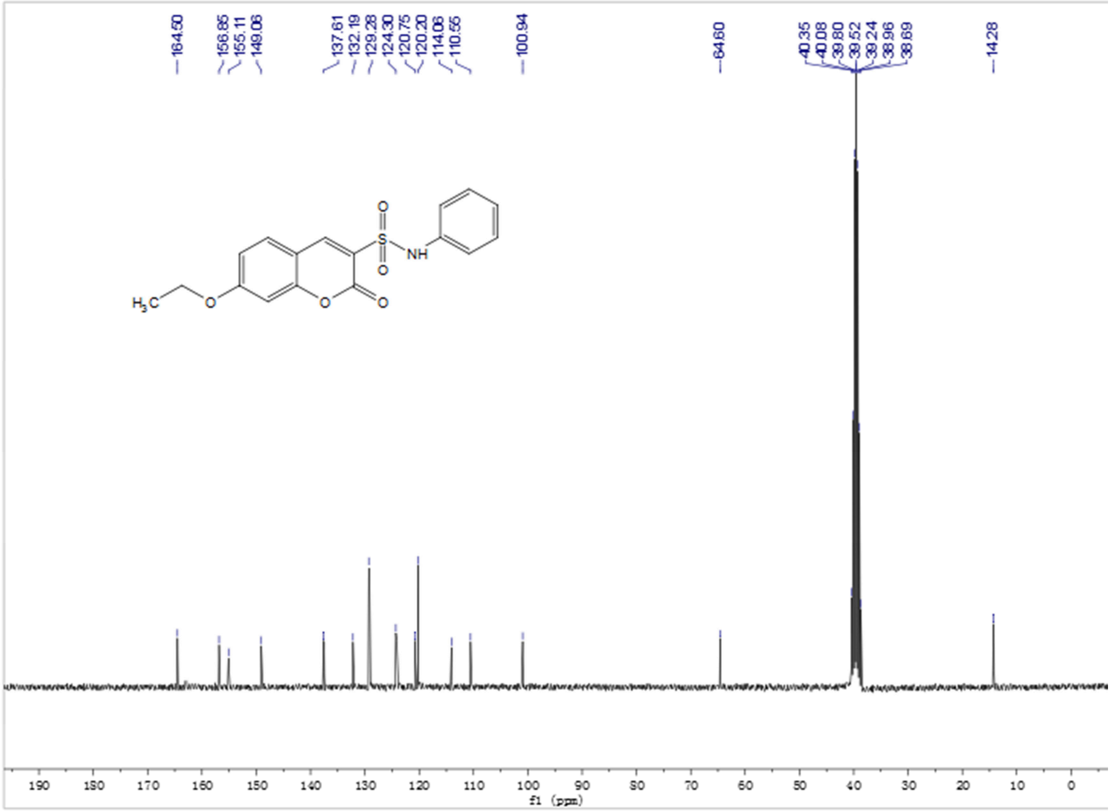

^{1}H NMR (300 MHz, $CDCl_3$) of Compound **7b**

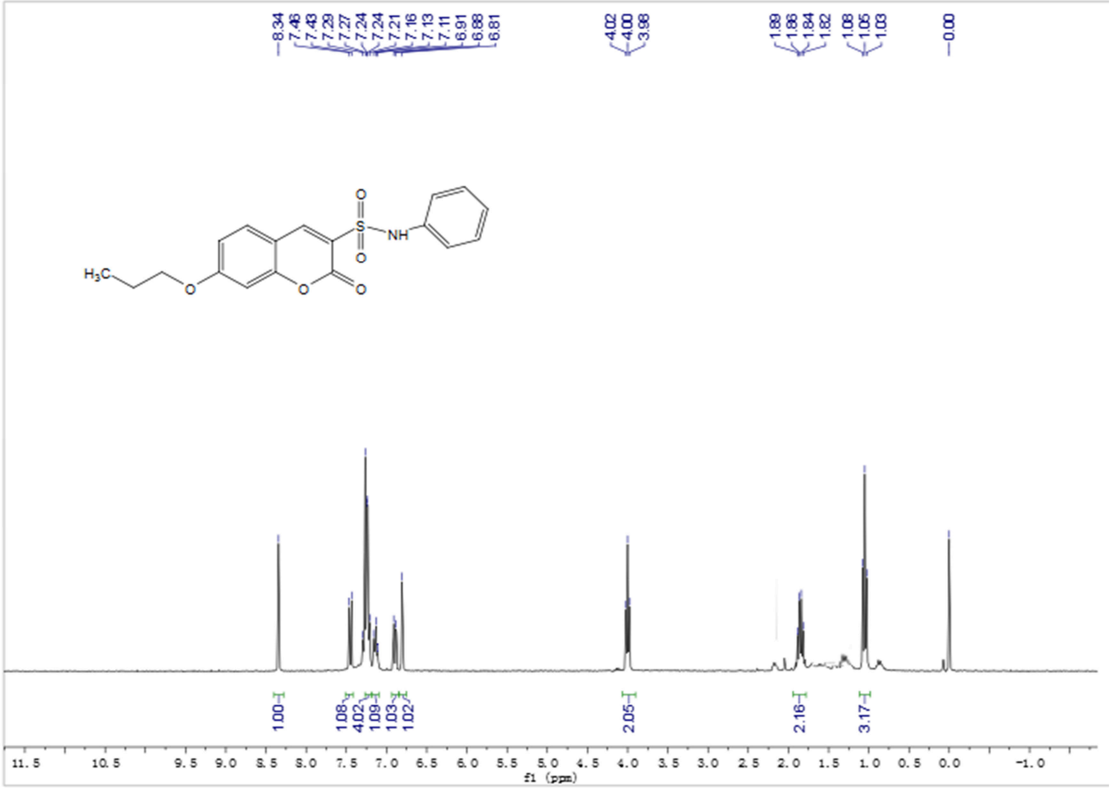

^{13}C NMR (75 MHz, DMSO-d_6) of Compound **7b**

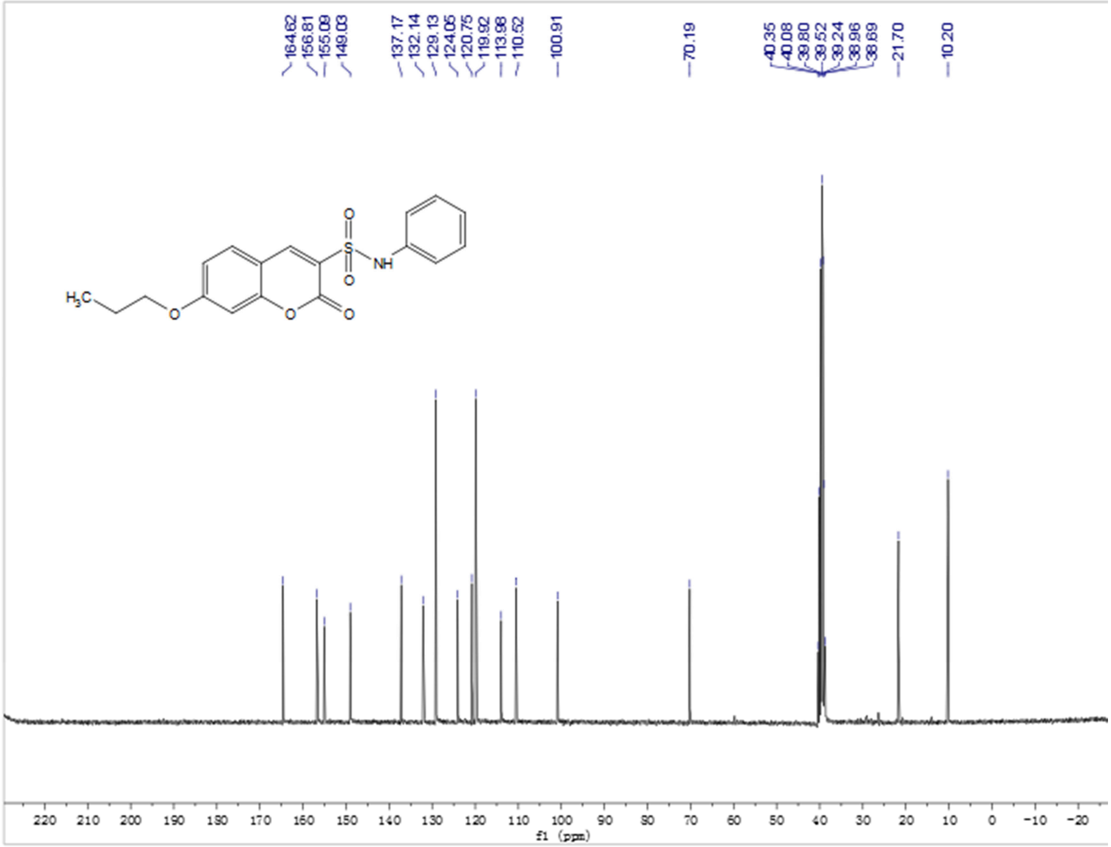

^{1}H NMR (300 MHz, DMSO-d_6) of Compound **9a**

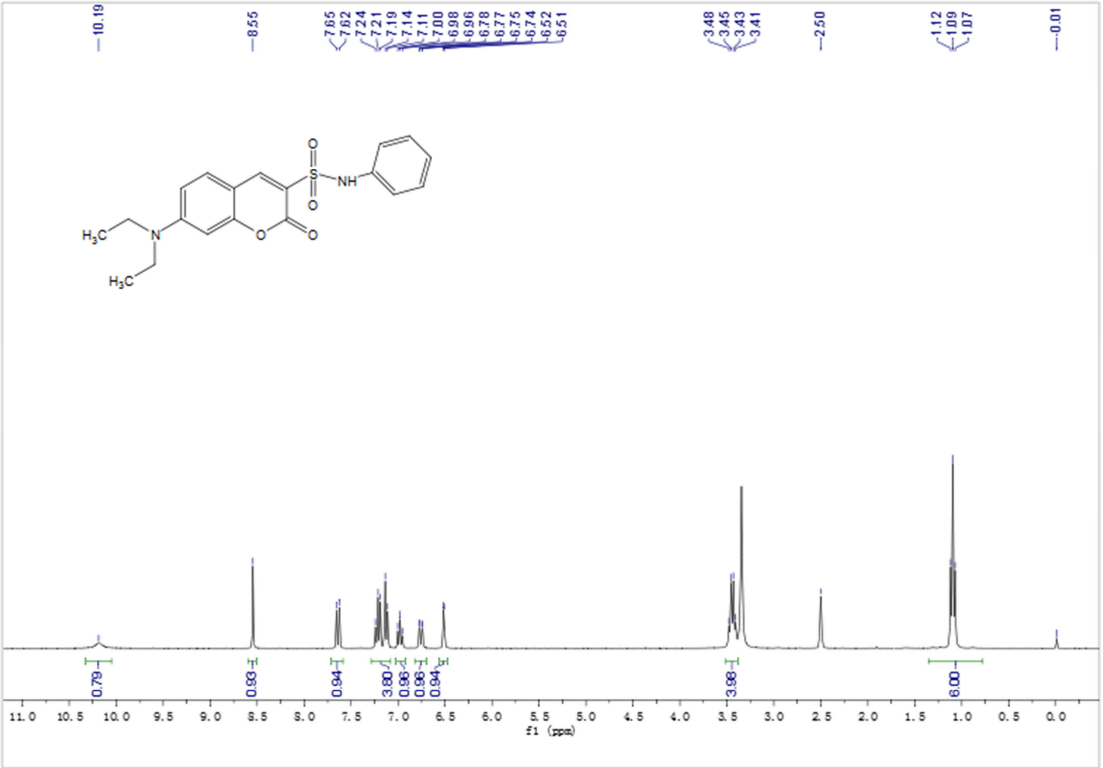

^{13}C NMR (75 MHz, DMSO-d_6) of Compound **9a**

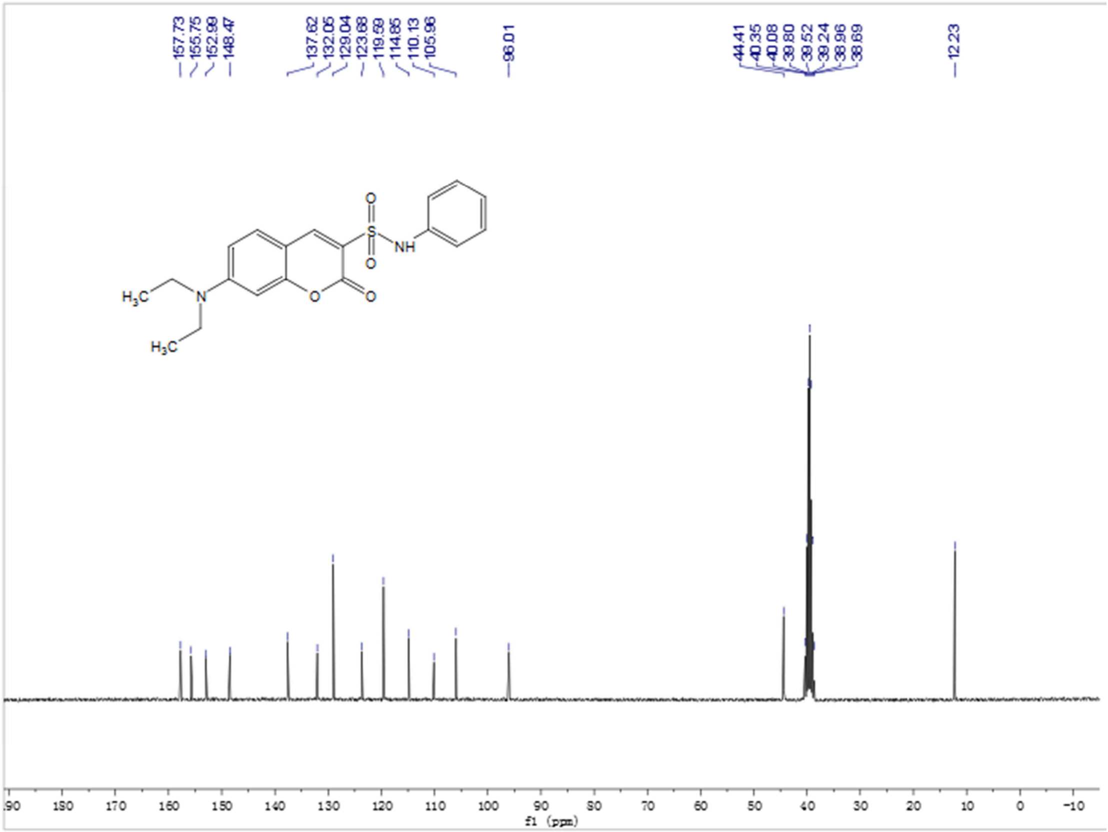

^{1}H NMR (300 MHz, $CDCl_3$) of Compound **9b**

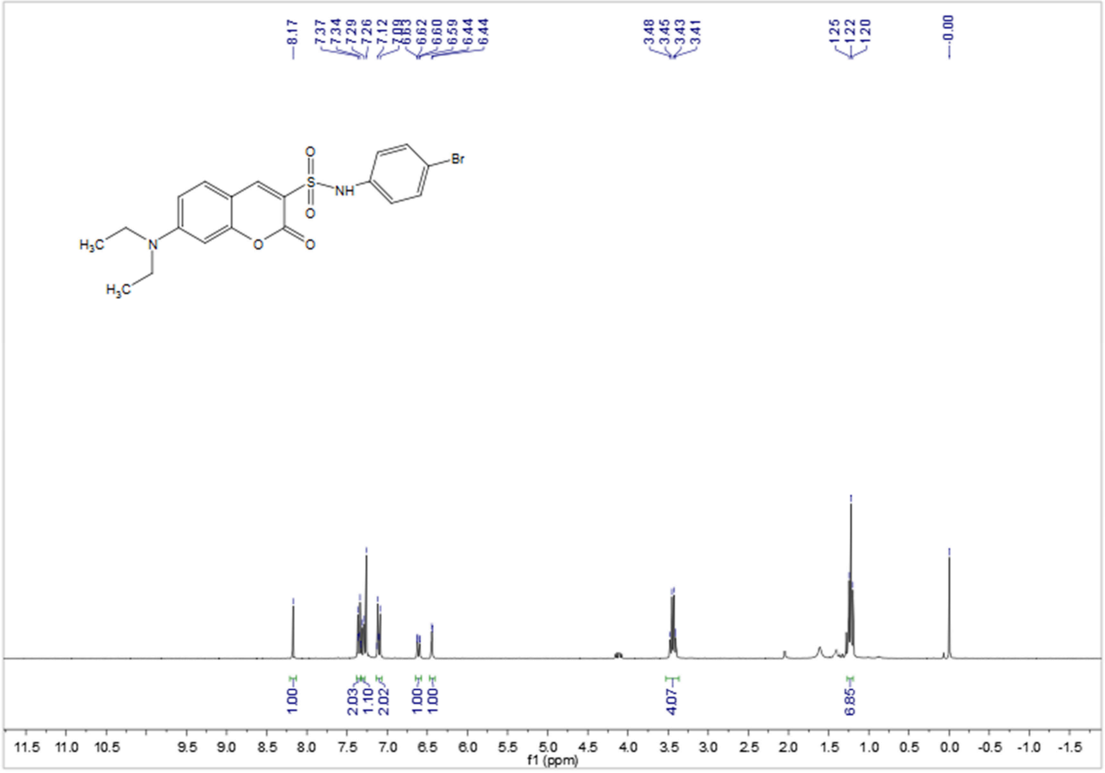

^{13}C NMR (75 MHz, DMSO-d_6) of Compound **9b**

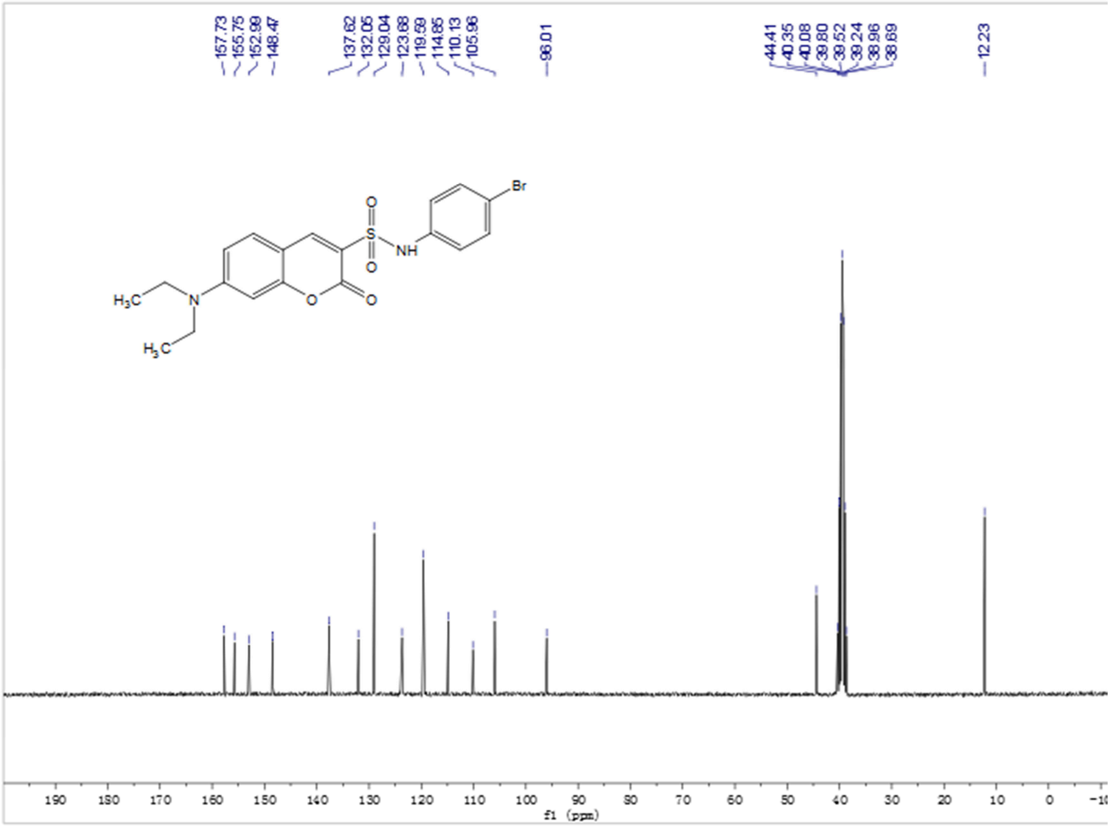

^{1}H NMR (300 MHz, $CDCl_3$) of Compound **9c**

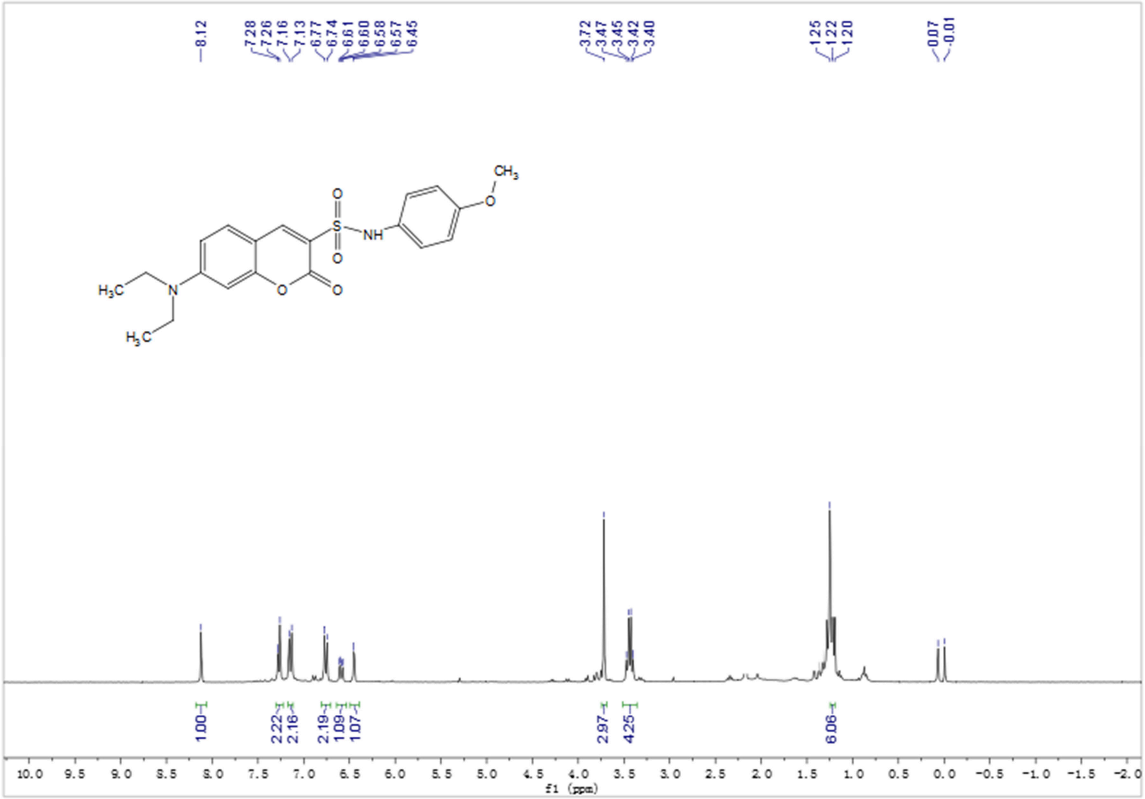

^{13}C NMR (75 MHz, DMSO-d_6) of Compound **9c**

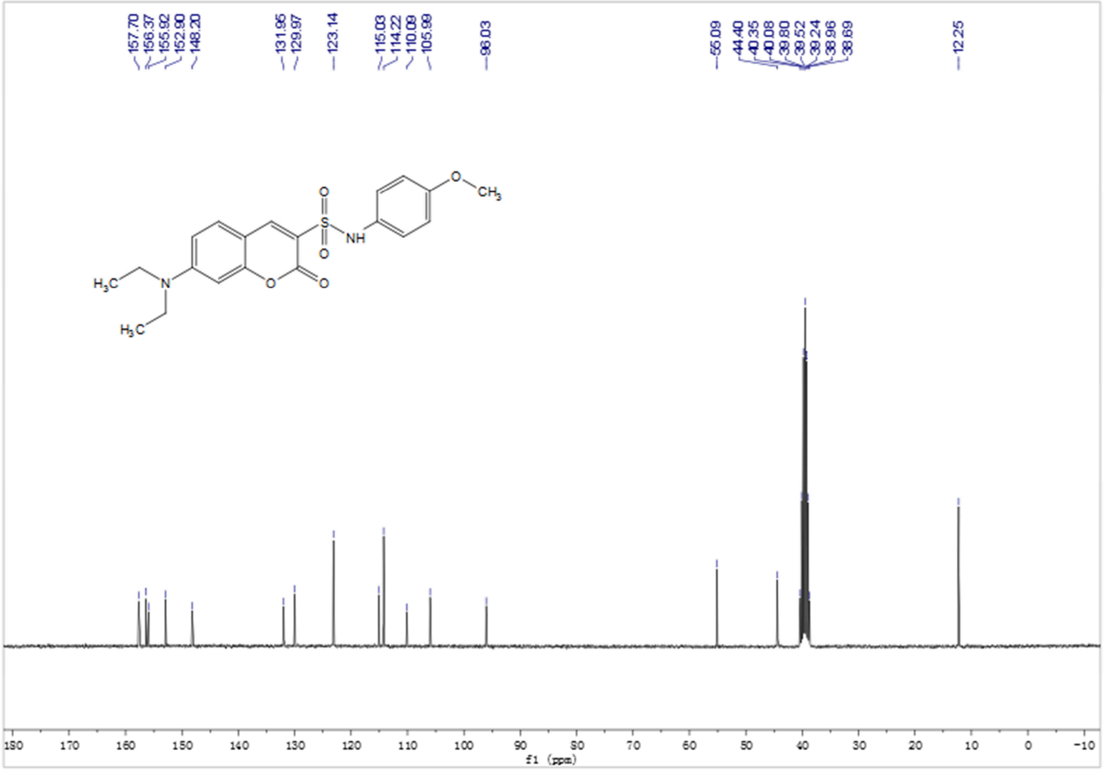

^{1}H NMR (400 MHz, $CDCl_3$) of Compound **11**

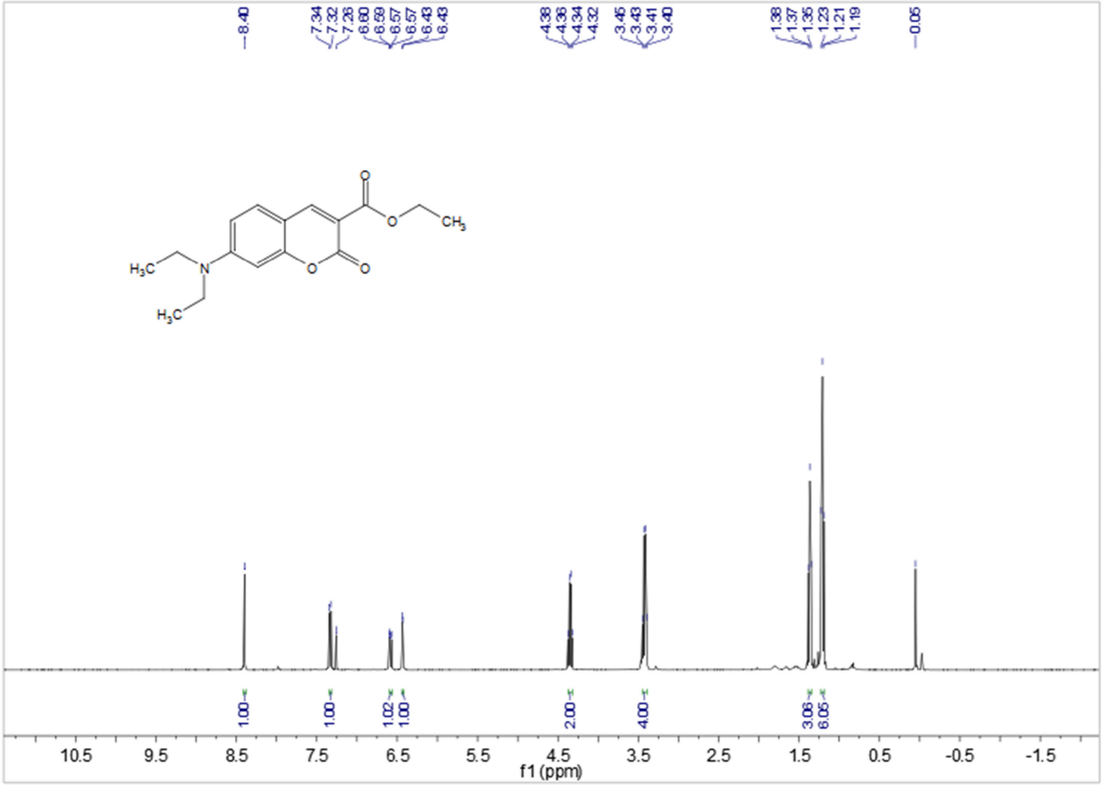

^{13}C NMR (100 MHz, $CDCl_3$) of Compound **11**

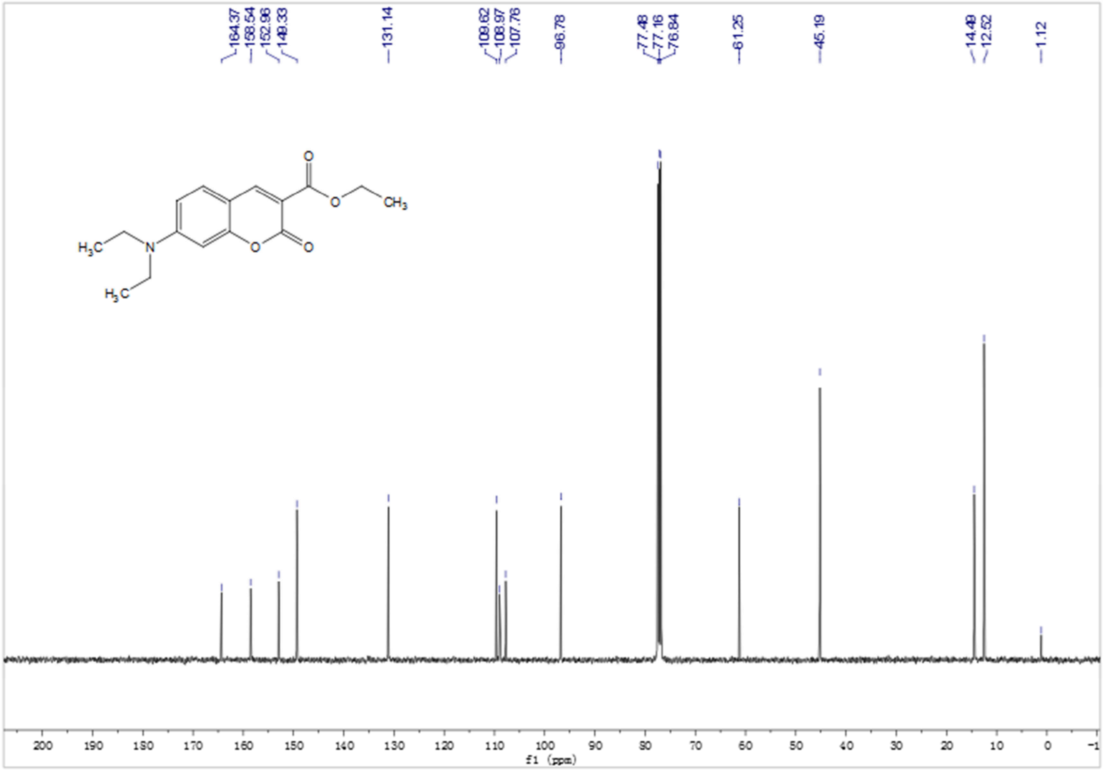

^{1}H NMR (400 MHz, $CDCl_3$) of Compound **12a**

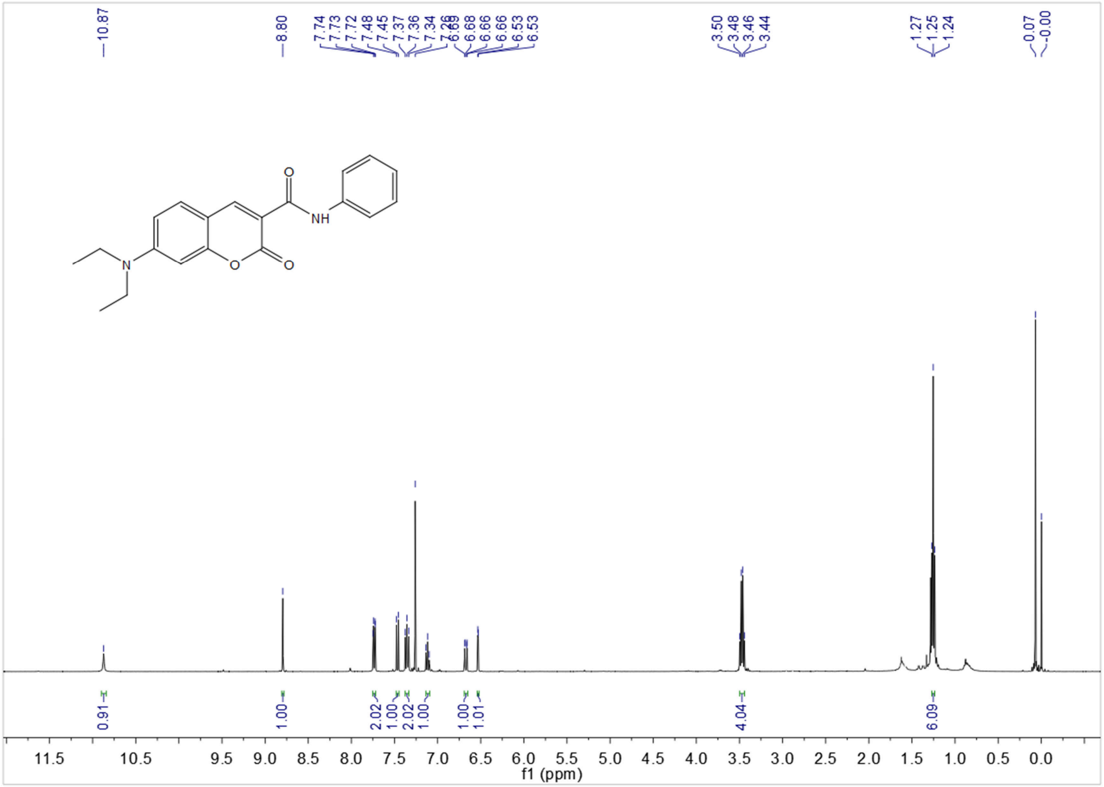

^{13}C NMR (100 MHz, $CDCl_3$) of Compound **12a**

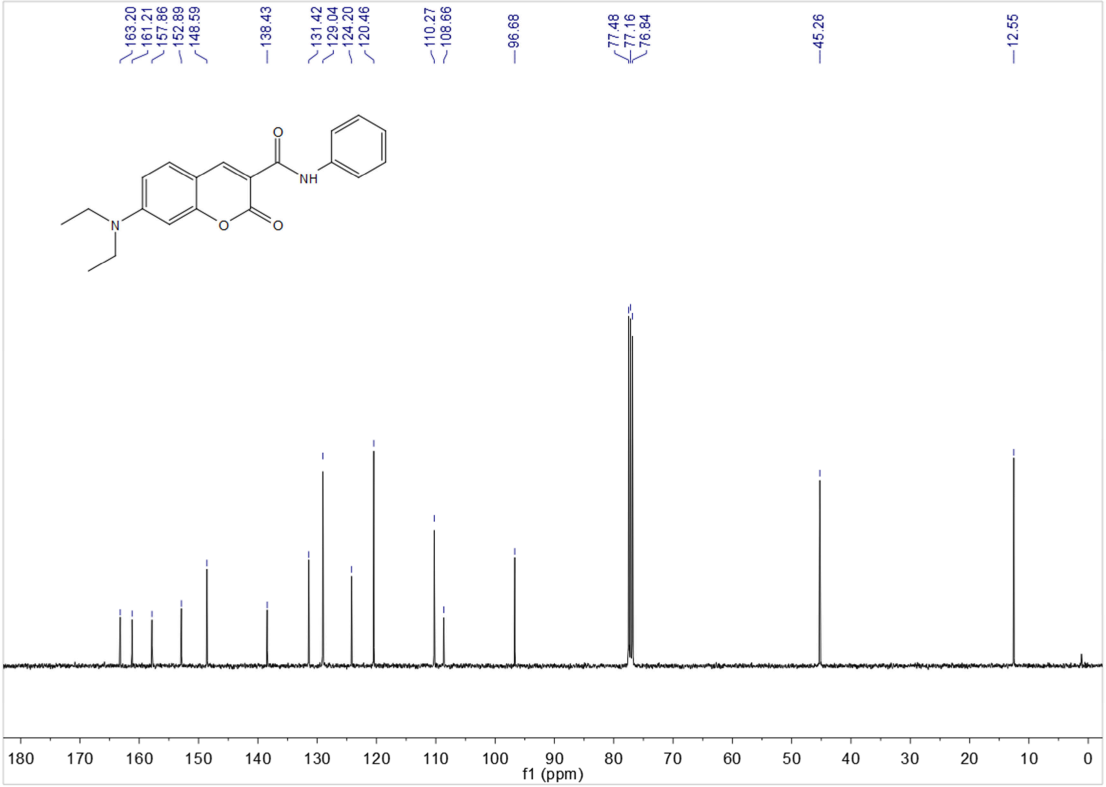

^{1}H NMR (400 MHz, $CDCl_3$) of Compound **12b**

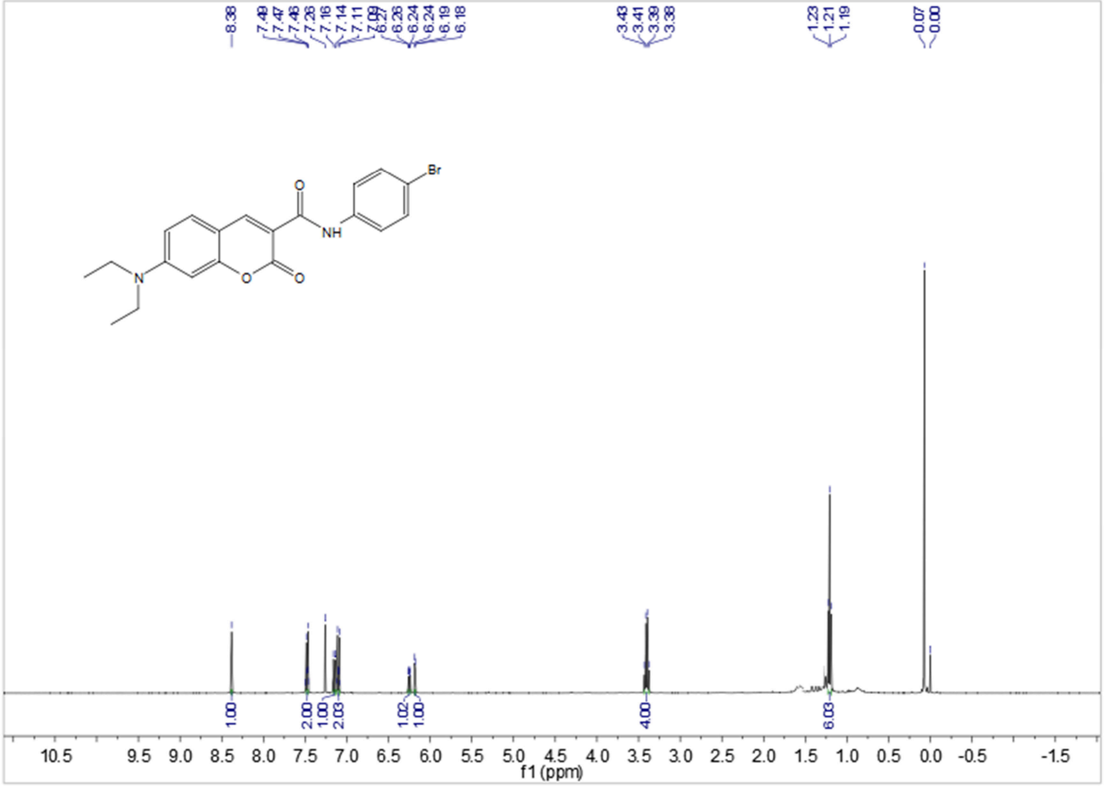

^{13}C NMR (100 MHz, $CDCl_3$) of Compound **12b**

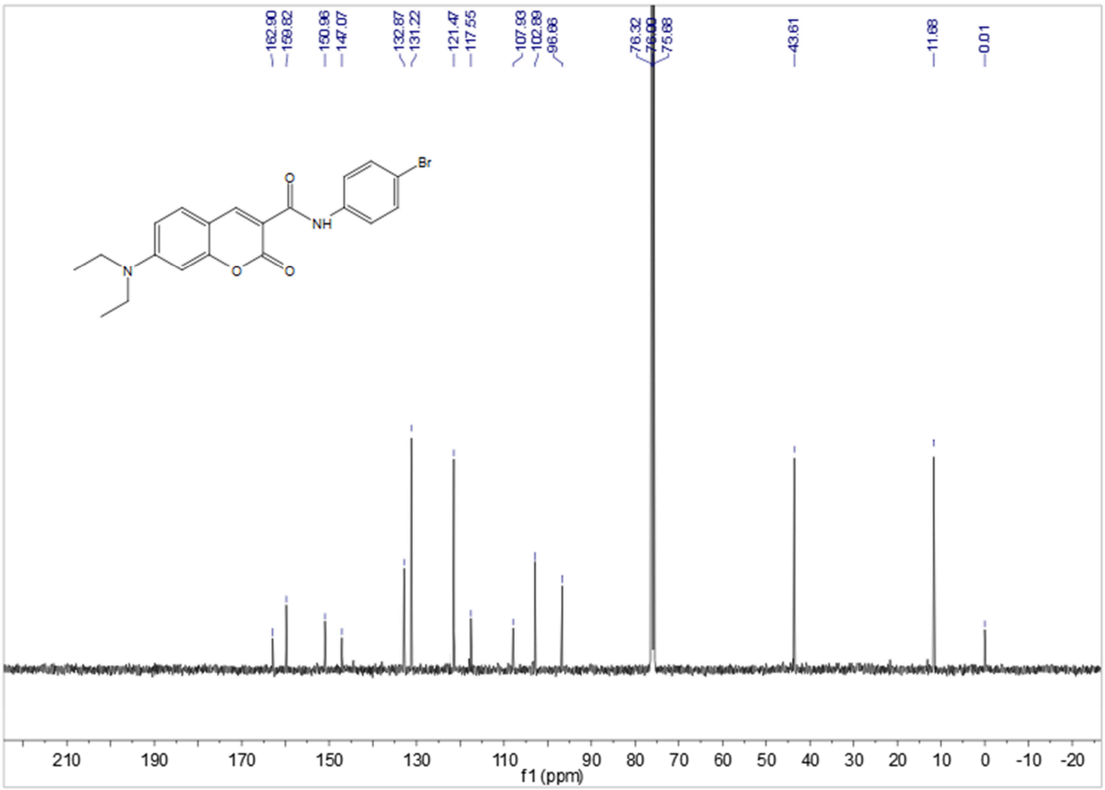

^{1}H NMR (400 MHz, $CDCl_3$) of Compound **12c**

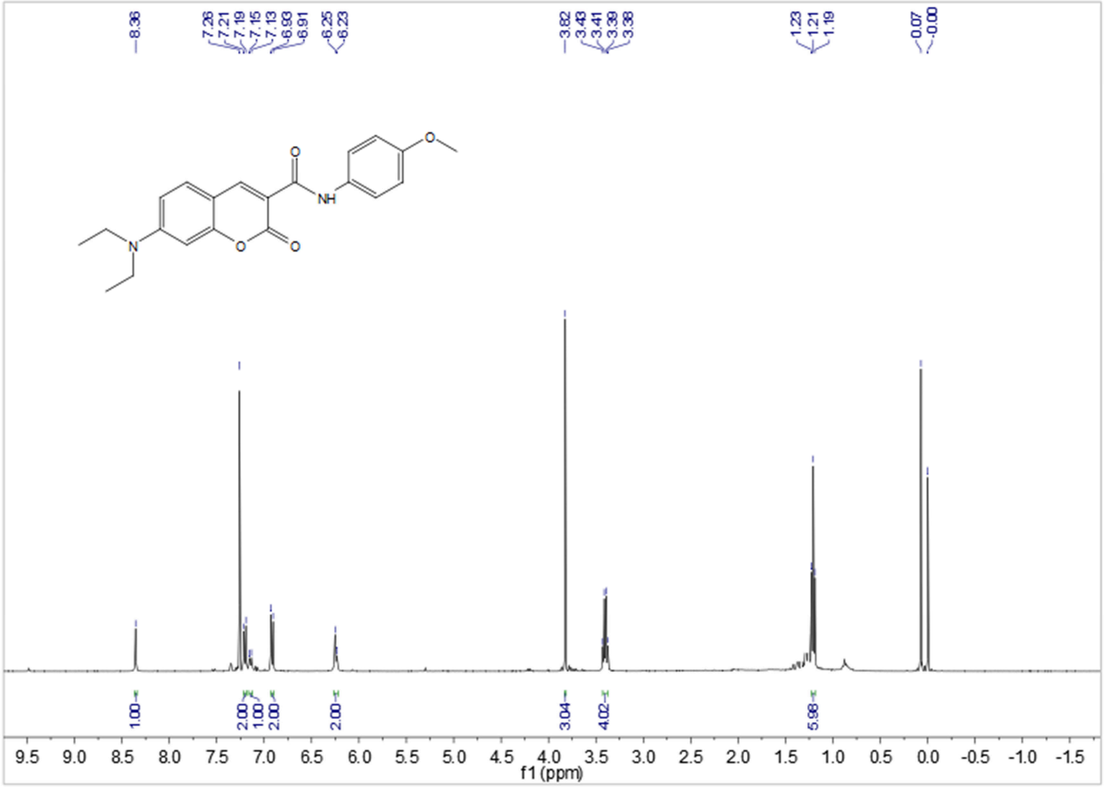

^{13}C NMR (100 MHz, $CDCl_3$) of Compound **12c**

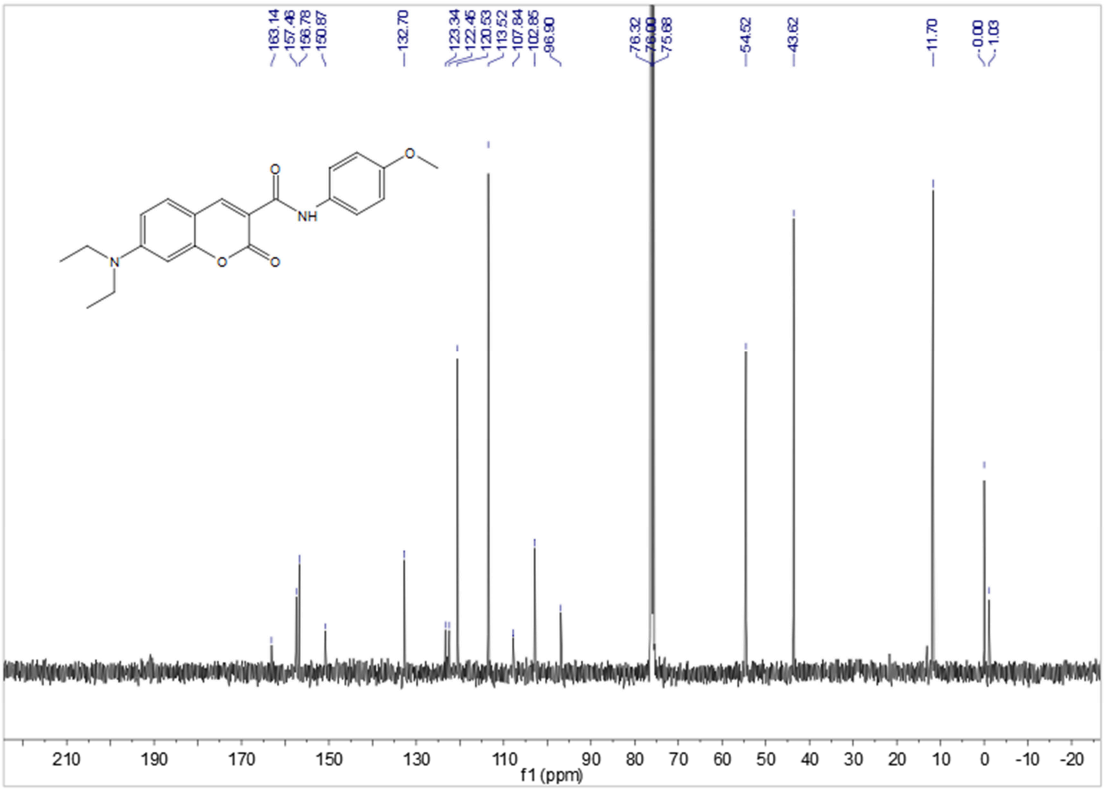
163.14
157.46
156.78
150.87
132.70
123.34
122.45
120.53
113.52
107.84
102.85
96.90
76.32
76.00
75.68
54.52
43.62
11.70
0.00
-1.03
O
NH
N
O
O
O
210 190 170 150 130 110 90 80 70 60 50 40 30 20 10 0 -10 -20
f1 (ppm)
